# Association of Urinary Complement Peptides with Kidney Function and Progression of Kidney Disease

**DOI:** 10.3390/ijms27041982

**Published:** 2026-02-19

**Authors:** Thi Minh Nghia Nguyen, Margarita Kondyli, Harald Mischak, Felix Keller, Joachim Beige, Agnieszka Latosinska, Justyna Siwy

**Affiliations:** 1 Mosaiques Diagnostics GmbH, 30659 Hannover, Germanymischak@mosaiques.de (H.M.);; 2 Institute for Molecular Cardiovascular Research (IMCAR), RWTH Aachen University Hospital, Pauwelsstrasse 30, 52074 Aachen, Germany; 3 Department of Internal Medicine IV (Nephrology and Hypertension), Medical University of Innsbruck, Anich St. 35, 6020 Innsbruck, Austria; 4 Kuratorium for Dialysis and Transplantation (KfH), 63263 Neu-Isenburg, Germany; 5 Department of Nephrology, Hospital St. Georg, 04129 Leipzig, Germany; 6 Department of Internal Medicine II, Martin-Luther-University Halle-Wittenberg, 06108 Halle (Saale), Germany

**Keywords:** complement, peptide, urine, biomarker, kidney disease, proteomics, CE-MS, personalized therapy

## Abstract

Complement activation has been implicated in many kidney diseases, but it remains unclear whether urinary complement-derived peptides reflect kidney function beyond albuminuria and predict disease progression. We analyzed mass spectrometry-based urinary peptidomics data from 10,939 individuals with chronic kidney disease and healthy controls. Fifty-eight complement-derived peptides were identified, predominantly from complement factor B (CFB) and C3. Of these, fifty-two were significantly related to estimated glomerular filtration rate (eGFR) independently of albuminuria, mostly inversely. Several C3- and CFB-derived peptides were also associated with specific kidney disease etiologies. In a longitudinal analysis of 3964 individuals (median follow-up 2.91 years), 18 of these peptides were significantly related to a major adverse kidney event (MAKE, defined as ≥40% eGFR decline, end-stage kidney disease or death) after adjustment for clinical covariates, indicating prognostic information beyond traditional risk markers. In the independent test cohort, combining these peptides in a machine learning-based model and adding the resulting risk score to clinical parameters significantly improved MAKE prediction (AUC 0.801 vs. 0.778, *p* = 0.031). Thus, urinary complement-derived peptides provide independent and clinically relevant information on kidney function and disease progression, supporting their potential value in the identification of high-risk patients and guiding more personalized therapy.

## 1. Introduction

Chronic kidney disease (CKD) is defined as abnormalities of kidney structure or function persisting for over three months, with significant health consequences [1]. It is a major and rapidly growing global health concern associated with significant health care costs, morbidity and mortality [2,3]. The Global Burden of Disease (GBD) consortium predicts CKD will become the fifth leading cause of death worldwide by 2040 and the second in countries with high life expectancy by the end of the century [4].

The most common causes of CKD are diabetes mellitus, hypertension, and glomerulonephritis [5]. When CKD progresses to end-stage kidney disease (ESKD), replacement therapy is required. CKD is typically diagnosed via reduced glomerular filtration rate (GFR) below 60 mL/min/1.73 m^2^ and/or the presence of pathological albuminuria (urinary albumin-to-creatinine ratio (UACR) ≥30 mg/g) [1,6]. Both parameters are the result of CKD, not the cause, and, as a consequence, only enable the detection of the disease at a late stage, when a significant proportion of organ function is lost.

The urinary peptidome, with its non-invasive collection, wide range and stability of peptides, and representation in extensive datasets for comparative studies [7], holds promise as a rich source for discovering CKD biomarkers that are involved in disease onset. Recent research has identified numerous urinary peptide biomarkers [8,9,10,11] that demonstrate significant potential for the early detection and stratification of kidney diseases. Among these promising avenues, complement-derived peptides have emerged as potential biomarkers for kidney diseases [11], as the dysregulation of the complement system is often involved in the tissue injury associated with CKD [12,13].

The complement system is activated through three distinct pathways, involving more than 30 plasma or membrane-bound proteins. The classical complement pathway is activated when immune complexes, such as IgG and IgM, bind to C1q. The mannose-binding lectin (MBL) pathway is initiated by microbial surface carbohydrates, recognized by MBL or other pattern recognition molecules like ficolins. In contrast, the alternative pathway undergoes continuous low-level activation (tick-over) and is further stimulated upon contact with various proteins, lipids, and carbohydrate structures on microbial or foreign surfaces [13,14]. Activation of these pathways leads to the formation of C3 convertases—C4b2a in the classical and lectin pathways and C3bBb in the alternative pathway—which continuously cleave C3 into C3a and C3b. The latter interacts with factor B, which is cleaved by factor D, to amplify C3 cleavage. This amplification step is central to the cascade, as C3 convertases further associate with additional C3b molecules to form C5 convertases (C4b2a3b and C3b2Bb), which cleave C5 into C5a and C5b. The subsequent binding of C5b to C6, C7, C8, and C9 results in the formation of the membrane attack complex (C5b-9, MAC), which perforates cell membranes, leading to the lysis of Gram-negative-like bacteria and aged erythrocytes. In nucleated cells, sublytic MAC pores can trigger cellular activation and tissue injury. Additionally, C3b functions as an opsonin, enhancing phagocytosis by binding to complement receptors such as CR1, CR2, CR3, and CR4 [13,14,15]. Complement system dysregulation contributes to inflammatory and autoimmune disorders, including kidney diseases like glomerulonephritis, atypical hemolytic uremic syndrome (aHUS), C3-glomerulopathy (C3G) [13,15], and other inflammatory kidney diseases like IgA nephropathy (IgAN) or ANCA-associated vasculitis [16]. This accumulation of evidence has resulted in the initiation of several clinical trials investigating the potential benefits of complement inhibitors for specific CKD etiologies [16,17]. The efforts to target complement activation in CKD would benefit substantially from specific complement-derived biomarkers guiding intervention and enabling assessment of therapeutic drug candidate impact.

Wendt et al. recently identified 23 urinary peptides derived from complement proteins C3, C4, and factor B (CFB). These peptides were also significantly linked to specific kidney disease etiologies, potentially indicating disease-specific complement activation [11]. In the present study, we aim to explore the urinary proteome with a particular focus on complement system-related peptides. By leveraging a comprehensive database of urinary peptides, we seek to identify novel biomarkers that inform on the potential involvement of the complement system in individual patients, and may in the future also guide intervention based on interference with complement activation.

## 2. Results

### 2.1. Study Population and Data Selection

**Figure 1:**
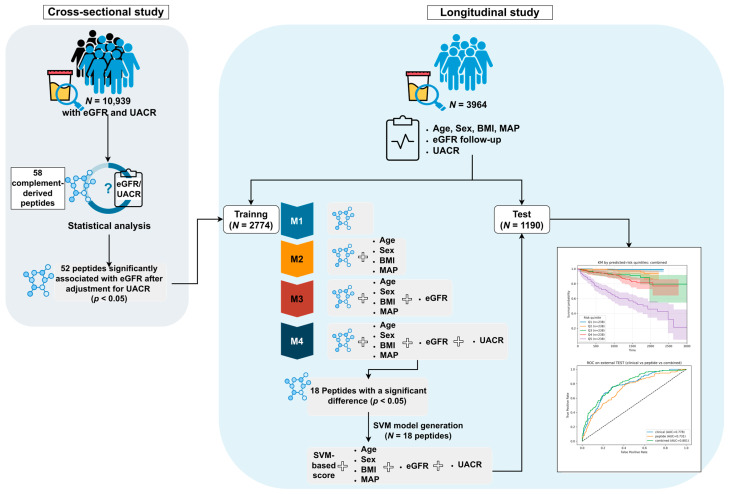
Overview of the study workflow. The study consisted of a cross-sectional and a longitudinal analysis. In the cross-sectional study, 58 complement-derived peptides were evaluated in a larger cohort (*N* = 10,939) for associations with eGFR, with and without adjustment for UACR. Peptides significantly associated with eGFR independent of UACR were carried forward. In the longitudinal cohort (*N* = 3964), a major adverse kidney event (MAKE: ≥40% eGFR decline, eGFR < 15 mL/min/1.73 m^2^, dialysis, kidney transplantation, or death) was assessed. The cohort was randomly split into a training (70%) and a test set (30%).

The study consisted of a cross-sectional and a longitudinal analysis as depicted in Figure 1. For the cross-sectional study, a total of 10,939 datasets with associated data on estimated GFR (eGFR) and UACR were extracted from the Human Urinary Proteome database for analysis [7]. These selected datasets were sourced from various previous studies [18,19,20,21,22,23,24,25,26,27,28,29,30]. In the longitudinal analysis, 3964 datasets were extracted from previously published studies [18,24,25,27,29,31,32]. For these datasets, complete information on sex, age, baseline and follow-up eGFR, and clinical covariates (body mass index (BMI), mean arterial pressure (MAP) and UACR) was available.

### 2.2. Cross-Sectional Study

Baseline characteristics of the study participants, including age, sex, eGFR, UACR, and underlying disease conditions, are summarized in Table 1. Stratified baseline characteristics by disease group are provided in Supplementary Table S1.

As shown in Table 1, in the cross-sectional study, 4699 (42.96%) participants were female, and 6240 (57.04%) were male, indicating a reasonably balanced sex distribution with a slight predominance of males. The cohort comprised both healthy controls and individuals with a broad spectrum of kidney diseases. The most frequent diagnosis was diabetic kidney disease (DKD, *N* = 4881; 44.62%), followed by unspecified kidney disease (CKD-others; *N* = 326; 2.98%) and autosomal dominant polycystic kidney disease (ADPKD; *N* = 265; 2.42%). Other kidney-related conditions were less common, including IgA nephropathy (IgAN; *N* = 88; 0.80%), focal segmental glomerulosclerosis (FSGS; *N* = 51; 0.47%), minimal change disease (MCD; *N* = 35; 0.32%), and other rare glomerular or systemic diseases.

The median age of the study population was 55 (interquartile range (IQR) 35–67) years. Median eGFR was 86.82 (IQR 61.43–114.58) mL/min/1.73 m^2^, indicating a wide range of kidney function across the cohort. The median UACR was 24.77 (IQR 7.58–430.41) mg/g, reflecting substantial variability in albuminuria. These baseline characteristics suggest a heterogeneous study population spanning a wide range of kidney function and albuminuria levels, making it well-suited to investigate complement-derived urinary peptides as markers of general kidney function rather than disease-specific alterations.

We were able to identify 58 different complement fragments in human urine using capillary electrophoresis (CE-) or liquid chromatography (LC-) coupled to tandem mass spectrometry (MS/MS). Of these, the majority were derived from CFB and C3, while fewer originated from complement factors D (CFD), C2, C4-A and C4-B. We first investigated, in the cross-sectional cohort, the associations of all 58 complement peptides with eGFR using Spearman’s rank correlation with and without adjustment for albuminuria status. Table 2 summarizes the unadjusted Spearman correlation coefficients (Rho) between each complement peptide and eGFR, along with the corresponding *p*-values, and the *p*-values for the peptide term from linear regression models of eGFR adjusted for UACR.

Most complement-derived peptides demonstrated a weak, yet significant, negative correlation with eGFR. All C2-, C4B-, and CFD-derived peptides were negatively correlated with eGFR, whereas C4A-, CFB- and C3-derived peptides displayed a heterogeneous pattern with both inverse and direct correlations. The most abundant C3-derived peptides (e010314, e019685, and e097631) showed a strong negative association with eGFR. In contrast, the two most abundant CFB-derived peptides (e097524 and e012507) were strongly positively associated with eGFR, whereas another CFB peptide (e207757) displayed a strong negative association with eGFR. After adjustment for UACR in multiple linear regression models, 52 of the 58 complement-derived peptides remained significantly associated with eGFR, confirming largely albumin-independent relationships. In most cases, effect sizes were attenuated, with C3- and CFB-derived peptides predominantly retaining inverse associations with eGFR.

**Figure 2:**
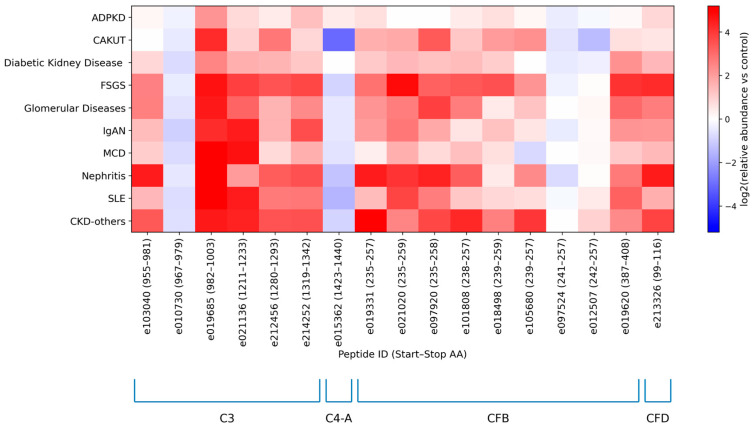
Log-transformed average relative abundances of significant complement-derived urinary peptides detected in >30% of samples, shown across disease groups and normalized to healthy controls. Peptide IDs are given as in Table 2 and are ordered by protein of origin: first C3, then C4-A, followed by complement factor B (CFB) and complement factor D (CFD). Within each protein, peptides are sorted according to the position of the first amino acid in the parent protein. The start and end amino acid positions of each peptide are indicated in parentheses next to the peptide ID. Abbreviations: Autosomal Dominant Polycystic Kidney Disease (ADPKD), Congenital Anomalies of the Kidneys and Urinary Tract (CAKUT), Focal Segmental Glomerulosclerosis (FSGS), IgA nephropathy (IgAN), Minimal Change Disease (MCD), Systemic Lupus Erythematosus (SLE).

To explore potential disease-specific patterns, we next examined the urinary excretion of the most frequently detected (frequency >30% of the total number of individuals) complement fragments stratified by disease etiology/condition with individual peptide excretion levels normalized to healthy controls (Table S2). Normalization was performed by dividing the mean peptide abundance for each disease/condition by the corresponding mean abundance in healthy controls. Figure 2 clearly shows that urinary excretion of most complement-derived peptides was higher in kidney disease than in healthy controls, with particularly pronounced increases in FSGS and other proteinuric glomerular diseases. Specifically, complement C3 peptides showed substantial increases, with the mean of normalized intensities ranging from 5.64 to 31.31, except for peptide e010730. Similarly, CFB-derived peptides were increased across disease groups (mean of normalized intensities 1.07–8.75), with the exception of peptide e097524. In contrast, the C4A fragment was lower in CKD than in controls (0.64), whereas the CFD fragment was markedly higher (7.87). In particular, peptides mapping to amino acid regions 982–1003, 1211–1233 and 1319–1342 of C3, 235–259 of CFB, and 99–116 of CFD were consistently up-regulated compared with controls. In contrast, peptides originating from the 967–979 region of C3, 1423–1440 region of C4-A and 241–257 region of CFB were predominantly down-regulated in CKD.

### 2.3. Longitudinal Study

Peptides that showed a significant association with eGFR in the cross-sectional analysis and remained independent of UACR were selected for further assessment in a longitudinal study. As shown in Table 3, the baseline median age was 60.00 (IQR 51.65, 67.00) years. 60.10% of the population were females. The median time of follow-up was 2.91 (IQR 2.07, 4.41) years. The baseline median eGFR was 83.90 (IQR 61.00, 96.88) mL/min/1.73 m^2^, suggesting that the cohort largely reflects early disease at study entry. Median BMI and MAP were 28.70 kg/m^2^ and 96.67 mmHg, respectively. Labeling of individuals as cases or controls was based on the MAKE definition described in the Materials and Methods. Overall, 447 composite events were observed among the 3964 individuals. Participants were randomly split into a training set (70%, *N* = 2774) for model development and a test set (30%, *N* = 1190) for independent evaluation, stratified by MAKE case/control status to preserve the case–control balance between datasets. Table 3 presents baseline characteristics for the training and test sets. The distributions of key clinical variables were similar between the two sets, indicating that the separation did not introduce meaningful imbalances in these clinical variables.

The associations between baseline complement-derived peptide intensities and the risk of MAKE were assessed in the training cohort using Cox proportional hazards regression models. Hazard ratios (HR) and 95% confidence intervals (CI) for all models are reported in Tables S3–S6. In univariate analyses (Model 1), forty-one peptides were significantly associated with MAKE risk. These associations remained robust after adjustment for demographic and baseline clinical covariates, including age, sex, BMI, and MAP (Model 2). Further adjustment for baseline kidney function (Model 3) attenuated some associations; however, a subset of twenty-one peptides remained significantly associated with MAKE. After additional adjustment for baseline UACR (Model 4), eighteen peptides remained significantly associated with MAKE risk. Notably, the majority of these peptides had previously shown inverse associations with eGFR in the cross-sectional analysis, consistent with their higher abundances being linked to more advanced kidney dysfunction. Particularly, the three most abundant C3-derived peptides (e010314, e019685 and e010730) and two most abundant CFB-derived peptides (e019331 and e207757) remained significantly associated with MAKE across all models (*p* = 0.031, 0.016, 0.003, 0.0001, and 0.003, respectively). In addition, a small number of peptides derived from complement C4-B and CFD also remained significantly associated with MAKE risk after full adjustment, although their effects were generally smaller than those observed for C3- and CFB-derived peptides.

**Figure 3:**
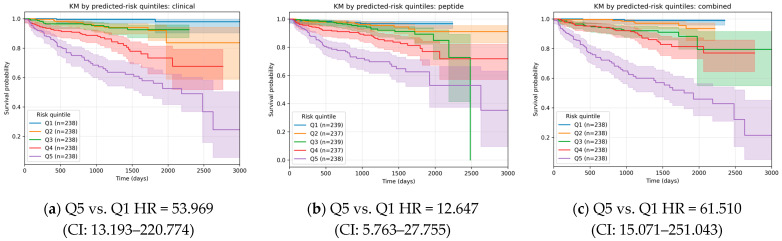
Kaplan–Meier survival curves stratified by predicted risk quintiles (Q1–Q5) with the corresponding confidence interval (CI) for (**a**) clinical, (**b**) peptidomics and (**c**) combined models in the test set.

**Figure 4:**
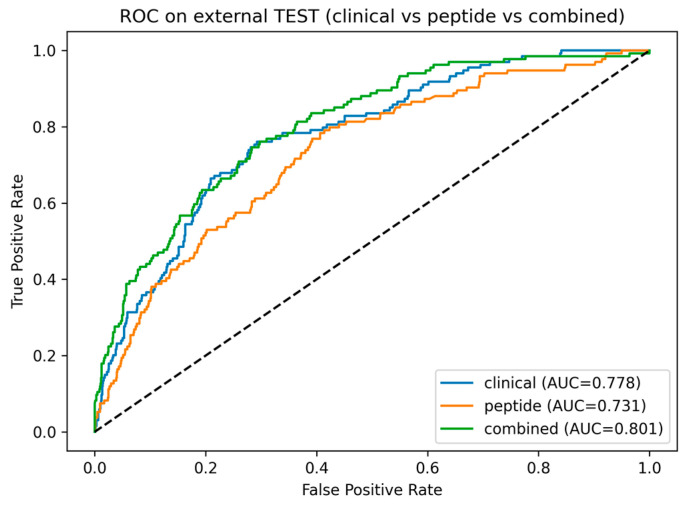
Receiver operating characteristic (ROC) curves and area under the characteristic curves (AUC) comparisons for clinical, peptidomics and combined models. Pairwise differences in AUC were assessed using DeLong’s test: clinical vs. peptidomics model (*p* = 0.052), combined vs. clinical model (*p* = 0.031), combined vs. peptidomics model (*p* = 0.0001).

Peptides that remained significant in the fully adjusted Cox model (*N* = 18, Model 4) were carried forward as candidate features for machine learning-based risk prediction. Support vector machine (SVM) models were trained and optimized in the training set (*N* = 2774 as shown in Figure 1) using peptidomics profiles, clinical variables, or their combination, and subsequently evaluated in the independent test set. In the test cohort, the model based on baseline clinical data only (clinical model) demonstrated good discrimination (area under the characteristic curves (AUC) = 0.778) and strong risk stratification across quintiles (HR = 53.969 for Q5 vs. Q1, Figure 3a). Adding peptide features to the model (combined model) with these baseline variables increased the AUC of the receiver operating characteristic (ROC) to 0.801 with HR 61.510 for Q5 vs. Q1 (Figure 3c). Pairwise comparisons using DeLong’s test (Figure 4) confirmed that the incorporation of peptide-derived features into the clinical model provided a statistically significant, albeit modest, improvement over the clinical-only model (*p* = 0.031). Consistent with this, a paired bootstrap comparison of the Cox trend slopes across quintiles showed a significantly steeper risk gradient for the combined model compared with the clinical model (*p* = 0.01). In a nested Cox model framework, adding the combined risk score to the clinical score significantly improved model fit (likelihood ratio test LR = 71.533, *p* = 0.0001), indicating that the combined score captured prognostic information beyond that contained in the clinical score alone.

## 3. Discussion

Complement activation is thought to play a central role in the inflammatory processes that drive kidney injury and disease progression. It induces the release of pro-inflammatory cytokines, which contribute to interstitial inflammation, a key feature in many forms of kidney damage [12,13,14]. Additionally, complement activation stimulates the production of extracellular matrix components, promoting fibrosis and scarring within the renal interstitium [13,33]. This, in turn, activates the renin-angiotensin system, further amplifying kidney damage by increasing blood pressure and contributing to hypertension [34]. These interconnected processes create a vicious cycle of injury, inflammation, and fibrosis, ultimately leading to kidney dysfunction and progression to ESKD. The initially generated cleavage products in the complement activation cascade are too large to freely pass through the glomerular filtration barrier (ranging between 15 and 50 kDa) [35]. However, complement activation results in the generation of smaller proteolytic fragments that can pass through the glomerular basement membrane and may appear in the urine. Alternatively, the urinary low-molecular-weight fragments may reflect intrarenal complement activation. The data available in this study do not allow distinguishing between these two alternatives.

In the present study, fifty-eight complement-derived urinary peptides were identified. Of these, fifty-two were significantly associated with eGFR, mostly with an inverse relationship and independent of albuminuria. This suggests that higher urinary complement fragment levels are associated with poorer kidney function and indicates that these peptides (predominantly, fragments of C3 and CFB) may capture complement-related pathophysiological processes beyond glomerular protein leakage.

Previous studies have shown that only minimal amounts of complement C3 are detectable in the urine of healthy individuals, whereas markedly increased urinary excretion is observed in IgAN and other glomerular diseases, including DKD and FSGS, and is associated with tubular injury [36,37,38,39]. Similarly, after adjustment for proteinuria, increased urinary excretion of C3 and CFB fragments has been reported across several kidney disease entities [11]. Consistent with these reports, we observed the highest excretion of abundant C3- and CFB-derived peptides in FSGS and other proteinuric glomerular disorders (Figure 2), compatible with enhanced glomerular passage and reduced proximal tubular reabsorption.

Notably, the peptides originating from C3 and CFD were consistently up-regulated across kidney disease etiologies, whereas peptides from C4-A were generally decreased. By contrast, CFB displayed a mixed, bidirectional pattern that varied by etiology, although most CFB-derived peptides were up-regulated overall. These changes were not uniform across all fragments but concentrated in specific peptide regions, arguing against a purely non-specific “protein leak” and instead suggesting selective complement processing. The fact that specific complement peptides can be consistently detected suggests that the peptides detected may be the terminal, stable products of complement activation, thereby providing an indirect measure of complement activity. The divergence observed between CKD etiologies further suggests that different etiologies may be associated with selective complement cleavage, generating pathway-dependent fragment profiles.

In longitudinal analyses, most peptides predicting MAKE showed the same direction of association as in cross-sectional analyses, linking higher urinary abundances to more advanced kidney dysfunction and higher subsequent event risk. While some associations were largely explained by baseline eGFR and UACR (e097631, e097524, and e012507), several C3- (e010314, e019685, e010730) and CFB-derived (e019331, e207757) peptides retained significant associations with outcome, indicating prognostic information beyond established risk markers.

In an independent test cohort, integrating peptide-derived features with clinical variables consistently improved model performance, supporting the robustness of these findings.

This study has several limitations. As an observational analysis, causality cannot be inferred. Elevated urinary complement peptides may reflect intrarenal activation, increased glomerular leakage with reduced tubular reabsorption, or both, and without paired plasma and kidney tissue data, their primary source cannot be determined. Moreover, the improvement in discrimination after adding peptide features to clinical models was modest, and its clinical utility requires further evaluation; additionally, residual confounding cannot be excluded despite adjustment for baseline eGFR and UACR.

Despite these limitations, our study also has certain strengths. While the study by Wendt et al. primarily provided a cross-sectional characterization of urinary complement-derived peptides across kidney disease entities and their relationship to kidney function and proteinuria, our work extends this framework to a longitudinal setting with clinically meaningful endpoints. By using a large, well-phenotyped cohort, applying stepwise adjustment for established risk factors, and validating prediction models in an independent test set, we demonstrate that a focused set of complement-derived peptides—particularly from C3 and CFB—captures prognostic information beyond routine clinical variables.

Building on these results, our findings also support a translational use case for integrating urinary peptidomics with clinical phenotyping to enable pathway-informed risk assessment and, ultimately, individualized treatments also in the context of therapeutic drugs targeting the complement system. While eGFR and UACR remain central for the detection of kidney impairment, they do not directly reflect underlying biologic processes that could be targeted by interventions. In contrast, the complement-derived peptide signature provides a mechanistic layer that may help identify patients in whom complement activity meaningfully contributes to ongoing injury. In complement-associated kidney diseases, such a signature could be exploited as an enrichment or stratification biomarker to identify patients most likely to benefit from complement-targeted therapies. This includes agents acting at a proximal complement or along the alternative and terminal pathways, such as iptacopan, pegcetacoplan, or other complement inhibitors [40,41,42]. In addition, complement-derived urinary peptides could serve as pharmacodynamic markers to monitor pathway modulation and biological response over time. Although our observational design cannot address treatment benefit directly, the consistent incremental prognostic information provided by the combined clinical–peptidomics model motivates prospective studies testing whether complement-peptide-guided stratification can improve trial design and support “treat-to-response” strategies in clinical practice.

## 4. Materials and Methods

### 4.1. Study Participants

This study included a cross-sectional discovery analysis and a longitudinal follow-up analysis. For the cross-sectional analysis, a total of 10,939 datasets from the Human Urinary Proteome Database [7] were used. The inclusion criteria were as follows: availability of eGFR (mL/min/1.73 m^2^) calculated using the CKD Epidemiology Collaboration (CKD-EPI) formula [43], information on UACR (mg/g) and availability of relevant demographic and clinical information such as age, sex, BMI, and MAP.

In the longitudinal analysis, CE-MS data from 3964 individuals drawn from previous studies with urine samples at the baseline visit were included in the analysis. Several covariables, including BMI, age, sex, MAP, eGFR, and UACR, were determined at the time of the baseline assessment. A MAKE was defined as (i) a reduction of ≥40% in eGFR during follow-up, with the date of this decline recorded as the event time; (ii) an eGFR value <15 mL/min/1.73 m^2^ at any point during follow-up, irrespective of the percentage decline; or (iii) initiation of dialysis or kidney transplantation, indicating progression to ESKD. (iv) In addition, if a participant died during follow-up without a previously documented CKD event, death was considered part of the composite outcome. Only one (the first) MAKE per individual was allowed, and if an endpoint was reached, the further endpoints were censored. The study was conducted according to the guidelines of the Declaration of Helsinki, and all datasets were fully anonymized. Ethical review and approval were not required for this study due to all data being fully anonymized, based on the opinion of the ethics committee of the Hannover Medical School, Germany (no. 3116-2016).

### 4.2. Sample Preparation, Capillary Electrophoresis–Mass Spectrometry (CE-MS) Analysis and Data Processing

Urinary proteome data were acquired based on CE-MS. Detailed descriptions of the CE-MS procedures, including reproducibility, repeatability, sample preparation, data evaluation, and normalization, have been previously reported [44]. Briefly, 0.7 mL of urine was thawed and diluted with 0.7 mL of a solution containing 2 M urea, 0.1 M NaCl, 10 mM NH4OH and 0.02% sodium dodecyl sulfate to suppress protein interactions. The sample was filtered and desalted using a PD 10 gel filtration column (GE Healthcare Bio Sciences, Uppsala, Sweden). Finally, the eluate was lyophilized, stored at 4 °C until the time of the CE-MS measurement, at which time samples were resuspended in 0.1 mL HPLC-grade H_2_O. The CE-MS analysis was performed with a P/ACE MDQ CE system (Beckman Coulter, Brea, CA, USA) coupled to a micro-TOF-MS (Bruker Daltonic, Bremen, Germany). Before each run, the capillary was rinsed with running buffer (2 min, 50 psi). Samples were injected at 2 psi for 99 s, corresponding to an injection volume of approximately 290 nL. Separation was performed at +25 kV for 30 min at 35 °C, followed by a pressure gradient (0.1–0.5 psi; 0.1–0.4 psi for 1 min each, then 0.5 psi for 30 min) while maintaining +25 kV. A sheath liquid (2-propanol/formic acid/water; 15 mL/200 µL/to 50 mL) was delivered coaxially at a flow rate of 0.02 mL/h without nebulizer gas. An electrospray ionization (ESI) was operated at −4.0 to −5.0 kV with the sprayer grounded. Mass spectra were acquired from m/z 400–3000 every 3 sec for approximately 60 min.

Mass spectral ion peaks corresponding to the same molecule but differing in charge state were deconvoluted into single masses using MosaFinder software [7] (version 2.5.1). Only signals with a charge state (z) >1, detected in at least three consecutive spectra, were retained for analysis. The resulting peak list characterized each polypeptide by its molecular mass and migration time. Calibration of mass and migration time was performed using 3151 internal standards, applying global linear regression for mass and local linear regression for migration time. To account for inter-sample variability of the obtained signal intensities, a normalization procedure was implemented using linear regression based on 29 collagen fragments, which are generally unaffected by disease and serve as internal standards [45]. The normalized intensity is referred to as the peptide abundance in this manuscript.

For identification of the amino acid sequences corresponding to the peptides detected by CE-MS, the acquired masses were compared with peptide sequences derived from CE- or LC-MS/MS analysis [46]. Analyses were carried out using either an Ultimate 3000 nano-flow LC system (Dionex Softron GmbH, Germering, Germany) or a P/ACE MDQ capillary electrophoresis system (Beckman Coulter, Brea, CA, USA), each coupled to a Q Exactive™ Plus mass spectrometer (Thermo Fisher Scientific Inc., Waltham, MA, USA). Orbitrap full-scan spectra were collected over m/z 300–2000, after which precursor ions were selected in a data-dependent manner for fragmentation.

Data files were searched against the UniProt human nonredundant database using Proteome Discoverer 2.4 and the SEQUEST HT search engine. Relevant settings were: no fixed modifications, oxidation of methionine and proline as variable modifications. Precursor mass tolerance was 5 ppm and fragment mass tolerance 0.05 Da.

### 4.3. Study Design and Statistical Analysis

In the cross-sectional analysis, the associations between all detectable complement-derived peptide fragments and eGFR in the selected cohort were evaluated using Spearman’s rank correlation on log-transformed peptide intensities (Scipy package, Python 3.14.2). In addition, using multiple linear regression, the UACR was included as an adjustment covariate to account for albuminuria status in models relating peptide intensities to eGFR; (UACR was retained on its original scale). Only peptides showing significant, UACR-independent associations (false discovery rate FDR-adjusted *p*-value < 0.05, Benjamini–Hochberg procedure) were carried forward to the longitudinal study. To investigate disease-specific patterns of complement activation, a heatmap was generated based on the most frequently detected complement fragments (frequency >30%), with urinary excretion normalized to healthy controls. Clinical diagnoses were harmonized into mechanistically coherent etiology groups to ensure adequate representation and biological interpretability.

In longitudinal analysis, the full cohort was randomly divided into a training set (70%) and a test set (30%), using stratification to preserve the distribution of CKD events in both subsets. The associations between baseline complement peptide intensities and the risk of a CKD event were examined using Cox proportional hazards regression models (lifelines package, Python). Both univariate and multivariable analyses were performed, and results are reported as HRs with 95% CIs.

These variables include the following:

- Model 1 (M1): peptide abundance to evaluate the association between CKD event and each peptide independently, serving as the baseline model;
- Model 2 (M2): peptide abundance and additional adjustments for demographic and clinical covariates, including age, sex, BMI, and MAP;
- Model 3 (M3): peptide abundance, age, sex, BMI, MAP, and eGFR to account for baseline kidney function;
- Model 4 (M4): peptide abundance, age, sex, BMI, MAP, eGFR, and incorporated UACR as an additional covariate to examine whether the peptide associations were independent of albuminuria.

The peptides that were identified as significant in model M4 from the Cox regression analyses were subsequently injected into a machine learning model. The SVM algorithm, including peptidomics profiles and clinical parameters, was trained separately and optimized to identify the best-performing set of peptides. Model performance was evaluated in the independent test set. Three models were validated: (i) a clinical model including only clinical covariates, (ii) a peptidomics model including only the selected complement-derived peptide features, and (iii) a combined model integrating both peptide features and clinical covariates. The peptidomics marker was developed using the SVM-based MosaCluster software [47] (version 1.7.0), yielding a single SVM-derived score that captures the selected complement-derived peptide features per patient. This score was then used as an input feature in the Python-based modeling workflow (scikit-learn), alongside the clinical covariates. The models were trained and evaluated using five-fold cross-validation in the model-building step. The parameters selected for the optimization (ranges specified in brackets) included: (a) the regularization strength C [0.1, 2, 200] and (b) the RBF kernel coefficient γ [2 × 10^−6^ to 2 × 10^−1^; 15 values spaced logarithmically]. To ensure comparable scaling across features, the SVM was implemented in a pipeline with z-score standardization (StandardScaler), and class imbalance was addressed using balanced class weights (class_weight = “balanced”). The optimization process involved acting on these parameters by testing how different values contribute to overall model performance in the independent validation set.

In the independent test set, the SVM-based risk score for each model was computed using the fixed model parameters. Kaplan–Meier curves for CKD events were generated across these risk-score quartiles (Q1–Q5). To discriminate between models, AUCs were compared using DeLong’s test for correlated ROC curves on the same test set. A paired bootstrap comparison of the Cox trend slopes across quintiles and a nested Cox model were also performed to further compare risk stratification performance. The study design is graphically depicted in Figure 1.

## 5. Conclusions

We show that complement-derived urinary peptides—particularly fragments originating from C3 and CFB—are consistently dysregulated across kidney disease etiologies and are strongly enriched in proteinuric/glomerular phenotypes. In a longitudinal setting, a subset of these peptides remained independently associated with MAKE after adjustment for baseline eGFR and UACR, and their integration with clinical variables provided a statistically significant, albeit modest, improvement in risk prediction. Together, these findings highlight urinary complement peptides as a promising tool to support more personalized management—by identifying patients with evidence of complement involvement who may be most likely to benefit from complement-targeted interventions and by providing a non-invasive readout to monitor biological response over time.

## Figures and Tables

**Table 1:** Demographic and clinical characteristics of the cross-sectional study population. Abbreviations: APDKD: autosomal dominant polycystic kidney disease; CAKUT: congenital anomalies of the kidney and urinary tract; FSGS: focal segmental glomerulosclerosis; IgAN: IgA nephropathy; MCD: minimal change disease; SLE: systemic lupus erythematosus; CKD-others: patients with compromised kidney function of unknown etiology and/or rare renal diseases not classified into the main diagnostic groups; eGFR: estimated glomerular filtration rate; UACR: urinary albumin-to-creatinine ratio.

Characteristic	N = 10,939
Gender (Female/Male)	4699/6240 (42.96%/57.04%)
APDKD	265 (2.42%)
CAKUT	55 (0.50%)
Diabetic Kidney Disease	4881 (44.62%)
FSGS	51 (0.47%)
Other Glomerular Diseases	53 (0.48%)
IgAN	88 (0.80%)
MCD	35 (0.32%)
Nephritis	14 (0.11%)
SLE	24 (0.22%)
CKD-others	326 (2.98%)
Healthy control	1442 (13.18%)
**Median (Q1, Q3) of characteristic**	
Age (years)	55 (35, 67)
eGFR (mL/min/1.73m^2^)	86.82(61.43, 114.58)
UACR (mg/g)	24.77(7.58, 430.41)

**Table 2:** Detected complement-derived urinary peptide fragments with the corresponding amino acid (AA) sequence, parental complement protein, peptide position in the protein sequence, average relative abundance of these peptides calculated based on the full dataset of 10,939 individuals, and the frequency of peptide detection. Unadjusted associations with eGFR are shown as Spearman’s rank correlation coefficient (Rho eGFR) with the corresponding *p*-value (*p*-Value eGFR). To account for albuminuria, we additionally fitted multivariable linear regression models with eGFR as the outcome and peptide intensity as the predictor, adjusting for UACR; the *p*-value for the peptide term is reported (*p*-Value eGFR|UACR). *p*-values were corrected for multiple testing using the false discovery rate (FDR).

Peptide ID	Sequence	Complement	Start AA	Stop AA	Avg. Rel. Abund.	Peptide Freq.	Rho eGFR	*p*-Value eGFR	*p*-Value eGFR|UACR
e007280	TNPTQKTKESL	C2	231	241	212.59	1440	−0.293	0	<0.0001
e097631	EGVQKEDIPPADLSDQVP	C3	955	972	1473.68	2019	−0.203	0	<0.0001
e102977	EGVQKEDIPPADLSDQVPDTESETRILLQ	C3	955	983	95.47	2590	0.114	<0.0001	<0.0001
e103040	EGVQKEDIPPADLSDQVPDTESETRIL	C3	955	981	214.69	4918	−0.118	0	<0.0001
e206120	EGVQKEDIPPADLSDQVPDTESETRILLQGTPVA	C3	955	988	40.30	611	−0.001	0.9758	0.2720
e010730	LSDQVPDTESETR	C3	967	979	1168.72	6677	0.089	<0.0001	<0.0001
e019685	LQGTPVAQMTEDAVDAERLKHL	C3	982	1003	2029.74	3565	−0.287	0	<0.0001
e018904	QGTPVAQMTEDAVDAERLKHL	C3	983	1003	268.48	1148	−0.196	<0.0001	<0.0001
e017878	GTPVAQMTEDAVDAERLKHL	C3	984	1003	192.31	1127	−0.191	<0.0001	<0.0001
e013736	QMTEDAVDAERLKHL	C3	989	1003	843.57	2487	−0.141	<0.0001	<0.0001
e214945	IAVHYLDETEQWEKFGLEKRQGALEL	C3	1023	1048	632.58	581	−0.066	0.1259	0.0951
e013669	LDETEQWEKFGLEK	C3	1028	1041	891.03	7896	0.054	<0.0001	0.0064
e209805	AFRQPSSA	C3	1058	1065	387.04	994	−0.122	0.0001	0.0002
e015560	EKQKPDGVFQEDAPVIH	C3	1110	1126	121.06	733	−0.123	0.0011	<0.0001
e012614	IGGLRNNNEKDMALT	C3	1130	1144	236.79	1791	−0.083	0.0005	<0.0001
e021136	LTTAKDKNRWEDPGKQLYNVEAT	C3	1211	1233	347.65	5369	−0.336	0	<0.0001
e010836	DKNRWEDPGKQL	C3	1216	1227	150.20	6258	0.032	0.0129	0.5193
e007220	NRWEDPGKQL	C3	1218	1227	215.52	2078	−0.189	0	<0.0001
e019876	MVFQALAQYQKDAPDHQELNL	C3	1274	1294	196.30	2157	−0.272	0	<0.0001
e203120	QALAQYQKDAPDHQELN	C3	1277	1293	406.52	3202	−0.198	0	<0.0001
e212456	AQYQKDAPDHQELN	C3	1280	1293	591.18	5282	−0.172	0	<0.0001
e004130	KDAPDHQEL	C3	1284	1292	97.71	1388	−0.265	0	<0.0001
e010314	ITHRIHWESASL	C3	1307	1318	3693.14	3183	−0.168	0	<0.0001
e011710	ITHRIHWESASLL	C3	1307	1319	296.30	479	0.047	0.3244	0.9836
e008838	THRIHWESASL	C3	1308	1318	125.89	1569	−0.164	<0.0001	<0.0001
e214252	LRSEETKENEGFTVTAEGKGQGTL	C3	1319	1342	538.49	6098	−0.254	0	<0.0001
e214673	ELNPLDHRGRTLEIPGNSDPNMIPDG	C4-A	947	972	73.99	3166	−0.060	0.0009	<0.0001
e011694	TKAPVDLLGVAHNNL	C4-A	1203	1217	723.11	1098	−0.186	<0.0001	<0.0001
e300114	LGVAHNNL	C4-A	1210	1217	164.60	1346	−0.232	0	<0.0001
e015362	DELPAKDDPDAPLQPVTP	C4-A	1423	1440	133.65	5351	0.182	0	<0.0001
e002966	QDEGAEPLK	C4-B	1157	1165	72.34	1843	−0.001	0.9756	0.0948
e011859	TKAPADLRGVAHNNL	C4-B	1203	1217	302.79	2150	−0.204	0	<0.0001
e204330	FLSSLTETIEGVDAEDGHGPGEQQ	CFB	234	257	242.00	1516	−0.426	0	<0.0001
e019331	LSSLTETIEGVDAEDGHGPGEQQ	CFB	235	257	1138.92	7464	−0.163	0	<0.0001
e021020	LSSLTETIEGVDAEDGHGPGEQQKR	CFB	235	259	136.68	3682	−0.296	0	<0.0001
e097920	LSSLTETIEGVDAEDGHGPGEQQK	CFB	235	258	382.31	6093	−0.368	0	<0.0001
e105938	LSSLTETIEGVDAEDGHGPGEQ	CFB	235	256	184.74	6479	−0.011	0.4034	0.0383
e208478	SSLTETIEGVDAEDGHGPGEQQ	CFB	236	257	471.82	2189	0.091	<0.0001	0.0004
e101808	LTETIEGVDAEDGHGPGEQQ	CFB	238	257	294.88	3719	−0.125	<0.0001	<0.0001
e018498	TETIEGVDAEDGHGPGEQQKR	CFB	239	259	130.58	4735	−0.156	0	<0.0001
e105680	TETIEGVDAEDGHGPGEQQ	CFB	239	257	575.33	3323	−0.171	0	<0.0001
e097524	TIEGVDAEDGHGPGEQQ	CFB	241	257	2613.28	10313	0.254	0	<0.0001
e012507	IEGVDAEDGHGPGEQQ	CFB	242	257	2459.53	8163	0.155	0	<0.0001
e005422	EGVDAEDGHGPG	CFB	243	254	270.67	3116	−0.025	0.1876	<0.0001
e008871	VDAEDGHGPGEQQ	CFB	245	257	330.31	1352	−0.311	0	<0.0001
e207757	KIVLDPSGSMN	CFB	260	270	1201.23	3211	−0.153	0	<0.0001
e211754	KIVLDPSGSMNIY	CFB	260	272	1752.00	1656	−0.027	0.3014	0.4724
e022704	YATYPKIWVKVSEADSSNADWVTKQL	CFB	314	339	101.42	944	−0.210	<0.0001	<0.0001
e009715	NEINYEDHKLK	CFB	340	350	143.08	2746	−0.179	0	<0.0001
e019620	MTDGLHNMGGDPITVIDEIRDL	CFB	387	408	207.36	6089	−0.143	0	<0.0001
e020254	MTDGLHNMGGDPITVIDEIRDLL	CFB	387	409	152.13	3048	−0.053	0.0044	0.0004
e017875	DGLHNMGGDPITVIDEIRDL	CFB	389	408	79.82	734	−0.251	<0.0001	<0.0001
e020614	TVDDKEHSIKVSVGGEKRDLEIE	CFB	529	551	172.44	1438	−0.280	0	<0.0001
e014441	PWLKEKLQDEDLGFL	CFB	750	764	918.51	1437	−0.311	0	<0.0001
e014598	ILGGREAEAHARPYMAS	CFD	26	42	422.27	2546	−0.075	0.0002	0.0014
e013024	RAVPHPDSQPDTIDH	CFD	99	113	211.35	1770	−0.044	0.0763	0.0204
e213095	RAVPHPDSQPDTIDHDL	CFD	99	115	933.81	2424	−0.322	0	<0.0001
e213326	RAVPHPDSQPDTIDHDLL	CFD	99	116	442.18	5073	−0.311	0	<0.0001

**Table 3:** Demographic and clinical characteristics of the longitudinal study population. Abbreviations: MAP: mean arterial pressure; BMI: body mass index; eGFR: estimated glomerular filtration rate; UACR: urinary albumin-to-creatinine ratio.

Characteristic	Overall (*N* = 3964)	Training (*N* = 2774)	Test (*N* = 1190)
Gender (Female/Male)	1581/2383 (39.90%/60.10%)	1101/1673 (39.69%/60.21%)	480/710 (40.34%/59.66%)
**Median (Q1, Q3) of characteristic**			
Duration of follow-up (years)	2.91 (2.07, 4.41)	2.90 (2.05, 4.39)	2.92 (2.10, 4.45)
Age (years)	60.00 (51.65, 67.00)	60.00 (51.13, 67.00)	60.00 (51.99, 67.00)
MAP (mmHg)	96.67 (90.98, 103.00)	96.67 (90.67, 103.00)	96.67 (91.00, 103.33)
BMI (kg/m^2^)	28.70 (25.7, 32. 44	28.70 (25.70, 32.45)	28.70 (25.70, 32.41)
eGFR (mL/min/1.73m^2^)	83.90 (61.00, 96.88)	84.17 (60.72, 97.12)	83.62 (61.23, 96.44)
UACR (mg/g)	9.85 (4.60, 32.45)	9.85 (4.66, 34.15)	9.58 (4.54, 28.41)

## Data Availability

Anonymized data are available from the corresponding author upon request. Proposals will be evaluated by the investigators and collaborators based on scientific merit. Upon approval, datasets will be provided via a secure online platform after execution of a data access and confidentiality agreement.

## Supplementary Materials

The following supporting information can be downloaded at: https://www.mdpi.com/article/10.3390/ijms27041982/s1.

## Abbreviations

The following abbreviations are used in this manuscript:

aHUS	Atypical Hemolytic Uremic Syndrome
APDKD	Autosomal Dominant Polycystic Kidney Disease
AUC	Area Under the characteristic Curves
BMI	Body Mass Index
C3G	C3-Glomerulopathy
CAKUT	Congenital Anomalies of the Kidney and Urinary Tract
CE	Capillary Electrophoresis
CFB	Complement Factor B
CFD	Complement Factor D
CI	Confidence Interval
CKD	Chronic kidney disease
CKD-EPI	CKD Epidemiology Collaboration
DKD	Diabetic Kidney Disease
eGFR	Estimated Glomerular Filtration Rate
ESKD	End-Stage Kidney Disease
FDR	False Discovery Rate
FSGS	Focal Segmental Glomerulosclerosis
GBD	Global Burden of Disease
GFR	Glomerular Filtration Rate
HR	Hazard Ratio
IgAN	IgA Nephropathy
LC	Liquid Chromatography
LR	Likelihood ratio
MAKE	Major Adverse Kidney Event
MAP	Mean Arterial Pressure
MCD	Minimal Change Disease
MS	Mass Spectrometry
ROC	Receiver Operating Characteristic
SLE	Systemic Lupus Erythematosus
SVM	Support Vector Machine
UACR	Urinary Albumin-to-Creatinine Ratio
