# Supplementary material for: Association of Urinary Complement Peptides with Kidney Function and Progression of Kidney Disease
Source: Int J Mol Sci. 2026 Feb 19;27(4):1982. doi: 10.3390/ijms27041982 (PMC12940597; doi:10.3390/ijms27041982)
Supplement: Supplementary file 1 [file ijms-27-01982-s001.zip › ijms-4125332-supplementary.pdf]

Supplementary information

Table S1: Baseline characteristics by disease group of the cross-sectional study population.Abbreviations:APDKD:autosomal dominant polycystic kidney disease; CAKUT: congenitalanomalies of the kidney and urinarytract;FSGS: focal segmental glomerulosclerosis; IgAN: IgA nephropathy; MCD:minimalchangedisease; SLE: systemic lupus erythematosus; CKD-others: patients with compromised kidney function of unknown etiology and/or rare renal diseases not classified into the main diagnostic groups.;eGFR:estimated glomerular filtration rate; UACR:urinary albumin-to-creatinine ratio.

Disease group	N (%)	Age, years (mean ± SD)	Female, n (%)	Male, n (%)	eGFR, mL/min/1.73m^2 (mean ± SD)	UACR (mg/g) (mean ± SD)
APDKD	265 (2.42%)	32.13 ± 8.17	147 (55.47%)	118 (44.53%)	92.46 ± 22.79	28.09 ± 36.21
CAKUT	55 (0.50%)	11.86 ± 3.41	16 (29.09%)	39 (70.91%)	26.97 ± 5.96	1066.95 ± 1106.80
Diabetic Kidney Disease	4881 (44.62%)	57.48 ± 14.79	2145 (43.94%)	2736 (56.05%)	84.18 ± 31.25	134.25 ± 738.60
FSGS	51 (0.47%)	51.61 ± 17.46	17 (33.33%)	34 (66.67%)	49.94 ± 31.33	2152.72 ± 2175.73
Glomerular Diseases	53 (0.48%)	48.30 ± 15.57	20 (37.74%)	33 (62.26%)	71.96 ± 33.77	3376.25 ± 3409.68
IgAN	88 (0.80%)	43.82 ± 14.62	22 (25.00%)	66 (75,00%)	60.03 ± 36.07	1273.04 ± 1464.86
MCD	35 (0.32%)	43.34 ± 17.58	11 (31.43%)	24 (68.57%)	76.76 ± 30.22	1633.14 ± 2220.21
Nephritis	14 (0.11%)	40.45 ± 26.61	7 (50.00%)	7 (50.00%)	29.70 ± 16.53	1438.64 ± 2092.45
SLE	24 (0.22%)	39.96 ± 11.90	18 (75.00%)	6 (25.00%)	71.75 ± 23.00	1299.66 ± 1759.68
CKD-others	326(2.98%)	61.65 ± 20.74	122 (37.42%)	204 (62.58%)	41.75 ± 27.28	1282.64 ± 1934.36
Healthy control	1442 (13.18%)	28.65 ± 10.13	652 (45.21%)	790 (54.79%)	124.96 ± 22.63	8.72 ± 22.63

Table S2: Log-transformed intensity of the most frequently detected (frequency > 30% of the total number of individuals) complement fragments normalized to healthy controls stratified by disease etiology/condition along with the corresponding mean value.

	C3						C4-A	CFB									CFD
	e103040	e010730	e019685	e021136	e212456	e214252	e015362	e019331	e021020	e097920	e101808	e018498	e105680	e097524	e012507	e019620	e213326
ADPKD	1.15	0.83	4.58	1.74	1.33	2.53	1.36	1.60	1.01	0.99	1.33	1.58	1.14	0.76	0.91	1.12	1.78
CAKUT	1.01	0.72	18.73	1.96	6.91	1.79	0.12	3.10	3.38	10.16	2.25	4.09	4.88	0.67	0.38	1.59	1.46
Diabetic Kidney Disease	1.80	0.59	5.65	3.09	2.87	2.23	1.01	2.17	2.77	2.41	2.67	2.05	0.98	0.72	0.78	4.73	2.81
FSGS	5.93	0.75	25.81	14.47	11.10	13.10	0.54	7.56	28.05	9.49	10.45	11.54	4.64	0.84	1.04	17.46	19.03
Glomerular Diseases	6.13	0.67	23.64	8.86	2.95	5.38	0.71	4.65	6.32	14.43	6.31	1.38	2.38	1.03	1.12	8.46	6.30
IgAN	2.61	0.50	18.21	22.46	3.07	12.09	0.70	4.20	6.79	3.36	1.52	2.46	1.48	0.74	1.06	4.53	4.36
MCD	2.06	0.59	35.86	26.50	1.73	3.14	0.66	1.29	3.15	1.71	2.50	1.60	0.57	1.01	1.11	2.16	2.69
Nephritis	22.41	0.70	38.73	4.13	10.04	11.27	0.42	22.76	16.95	20.92	9.60	1.38	5.38	0.60	1.05	6.43	23.32
SLE	2.81	0.62	117.91	22.58	6.44	6.93	0.34	2.61	13.43	6.39	2.24	1.76	1.65	0.91	1.37	9.18	3.10
Others	10.45	0.63	24.02	21.12	11.22	11.31	0.55	33.63	5.72	12.73	19.70	6.13	16.10	1.00	1.91	5.40	13.93
Mean	5.64	0.66	31.31	12.69	5.77	6.98	0.64	8.36	8.76	8.26	5.86	3.40	3.92	0.83	1.07	6.11	7.88

Table S3: Associations between baseline complement peptide intensities and the risk of a CKD event were examined using Cox proportional hazards regression model in training cohort (N=2,774): Model 1

Predictor	Hazard_Ratio	CI_Lower	CI_Upper	P-Value
e097920	1.365	1.302	1.430	p<0.0001
e019685	1.210	1.174	1.248	p<0.0001
e097631	1.174	1.134	1.216	p<0.0001
e017875	1.318	1.235	1.407	p<0.0001
e015362	0.802	0.761	0.846	p<0.0001
e014441	1.178	1.132	1.226	p<0.0001
e021136	1.209	1.150	1.270	p<0.0001
e019876	1.255	1.182	1.332	p<0.0001
e097524	0.833	0.790	0.878	p<0.0001
e008838	1.222	1.153	1.295	p<0.0001
e013669	0.881	0.847	0.916	p<0.0001
e101808	1.220	1.145	1.301	p<0.0001
e213326	1.147	1.097	1.201	p<0.0001
e203120	1.143	1.093	1.195	p<0.0001
e010314	1.115	1.073	1.159	p<0.0001
e014598	1.123	1.077	1.171	p<0.0001
e022704	1.309	1.184	1.447	p<0.0001
e011694	1.184	1.109	1.264	p<0.0001
e103040	1.155	1.092	1.222	p<0.0001
e021020	1.157	1.092	1.226	p<0.0001
e214252	1.124	1.072	1.179	p<0.0001
e010730	0.918	0.886	0.951	p<0.0001
e204330	1.426	1.231	1.652	p<0.0001
e008871	1.197	1.111	1.291	p<0.0001
e212456	1.118	1.065	1.172	p<0.0001
e007280	1.182	1.090	1.283	p<0.0001
e013736	1.100	1.049	1.155	p<0.0001
e019331	1.102	1.046	1.161	2.76e-04
e020614	1.116	1.051	1.184	3.50e-04
e005422	0.867	0.788	0.954	3.52e-03
e012614	0.866	0.785	0.954	3.72e-03
e011859	1.107	1.033	1.185	3.78e-03
e213095	1.097	1.028	1.171	5.48e-03
e009715	1.077	1.019	1.137	8.57e-03
e209805	0.799	0.673	0.948	1.00e-02
e105680	1.080	1.015	1.149	1.46e-02
e207757	1.056	1.008	1.106	2.20e-02
e208478	1.111	1.012	1.220	2.64e-02
e015560	1.173	1.018	1.352	2.69e-02
e300114	0.871	0.767	0.988	3.14e-02
e105938	1.059	1.003	1.119	3.99e-02
e013024	0.920	0.840	1.007	7.19e-02
e018904	1.078	0.958	1.212	2.11e-01
e017878	1.091	0.944	1.261	2.38e-01
e019620	1.031	0.973	1.093	3.04e-01
e018498	0.973	0.908	1.043	4.40e-01
e004130	0.979	0.867	1.104	7.28e-01
e012507	1.007	0.960	1.056	7.80e-01
e214673	1.006	0.926	1.093	8.84e-01

Predictor	Hazard_Ratio	CI_Lower	CI_Upper	P-Value
e007220	1.007	0.916	1.106	8.88e-01
e020254	0.996	0.935	1.061	8.95e-01
e102977	0.996	0.924	1.073	9.08e-01

Table S4: Associations between baseline complement peptide intensities and the risk of a CKD event were examined using Cox proportional hazards regression model in training cohort (N=2,774): Model 2

Predictor	Hazard_Ratio	CI_Lower	CI_Upper	P_Value
e300114	0.859	0.757	0.975	1.84e-02
Age	1.048	1.034	1.062	p<0.0001
Sex	1.385	1.095	1.751	6.56e-03
BMI	0.962	0.936	0.990	6.99e-03
MAP	1.021	1.007	1.034	2.27e-03
e209805	0.794	0.669	0.943	8.51e-03
Age	1.047	1.033	1.061	p<0.0001
Sex	1.381	1.092	1.747	7.12e-03
BMI	0.963	0.937	0.990	7.86e-03
MAP	1.021	1.008	1.035	1.67e-03
e004130	0.967	0.855	1.094	5.94e-01
Age	1.047	1.033	1.062	p<0.0001
Sex	1.382	1.093	1.747	6.93e-03
BMI	0.963	0.936	0.990	7.99e-03
MAP	1.021	1.008	1.035	1.74e-03
e005422	0.846	0.769	0.930	5.55e-04
Age	1.048	1.034	1.062	p<0.0001
Sex	1.420	1.123	1.795	3.39e-03
BMI	0.963	0.937	0.990	8.17e-03
MAP	1.021	1.008	1.035	2.11e-03
e207757	1.073	1.025	1.124	2.57e-03
Age	1.048	1.034	1.062	p<0.0001
Sex	1.408	1.112	1.783	4.44e-03
BMI	0.961	0.934	0.988	4.85e-03
MAP	1.021	1.008	1.034	1.33e-03
e007220	1.018	0.928	1.116	7.04e-01
Age	1.047	1.033	1.062	p<0.0001
Sex	1.382	1.092	1.750	7.09e-03
BMI	0.962	0.935	0.989	6.76e-03
MAP	1.021	1.008	1.035	1.77e-03
e007280	1.157	1.066	1.256	4.84e-04
Age	1.046	1.032	1.060	p<0.0001
Sex	1.368	1.081	1.731	9.21e-03
BMI	0.961	0.935	0.988	5.37e-03
MAP	1.022	1.009	1.036	1.01e-03
e008838	1.210	1.139	1.285	p<0.0001
Age	1.047	1.033	1.061	p<0.0001
Sex	1.340	1.057	1.697	1.54e-02
BMI	0.960	0.935	0.987	3.28e-03
MAP	1.019	1.007	1.032	3.03e-03
e008871	1.183	1.101	1.271	p<0.0001
Age	1.047	1.033	1.061	p<0.0001

Predictor	Hazard_Ratio	CI_Lower	CI_Upper	P_Value
Sex	1.382	1.093	1.747	6.89e-03
BMI	0.961	0.935	0.989	5.89e-03
MAP	1.021	1.008	1.034	1.50e-03
e009715	1.065	1.007	1.125	2.71e-02
Age	1.047	1.032	1.061	p<0.0001
Sex	1.370	1.083	1.732	8.59e-03
BMI	0.960	0.934	0.988	4.69e-03
MAP	1.022	1.008	1.035	1.76e-03
e010314	1.137	1.091	1.185	p<0.0001
Age	1.050	1.036	1.064	p<0.0001
Sex	1.356	1.071	1.718	1.16e-02
BMI	0.961	0.935	0.988	4.99e-03
MAP	1.018	1.005	1.031	6.45e-03
e010730	0.915	0.883	0.948	p<0.0001
Age	1.047	1.034	1.061	p<0.0001
Sex	1.363	1.077	1.724	1.00e-02
BMI	0.965	0.938	0.992	1.12e-02
MAP	1.021	1.008	1.035	1.66e-03
e011694	1.186	1.107	1.271	p<0.0001
Age	1.048	1.034	1.062	p<0.0001
Sex	1.324	1.046	1.676	1.96e-02
BMI	0.960	0.934	0.987	3.73e-03
MAP	1.021	1.008	1.034	1.73e-03
e011859	1.105	1.030	1.185	5.14e-03
Age	1.047	1.033	1.062	p<0.0001
Sex	1.361	1.076	1.720	1.01e-02
BMI	0.960	0.933	0.987	4.00e-03
MAP	1.022	1.008	1.035	1.42e-03
e012507	1.024	0.974	1.078	3.50e-01
Age	1.048	1.033	1.062	p<0.0001
Sex	1.398	1.104	1.771	5.39e-03
BMI	0.963	0.936	0.990	7.47e-03
MAP	1.021	1.008	1.035	1.71e-03
e212456	1.094	1.043	1.147	2.33e-04
Age	1.046	1.032	1.060	p<0.0001
Sex	1.321	1.042	1.675	2.15e-02
BMI	0.962	0.936	0.989	6.41e-03
MAP	1.021	1.008	1.035	1.73e-03
e013024	0.930	0.848	1.019	1.20e-01
Age	1.047	1.033	1.061	p<0.0001
Sex	1.405	1.111	1.776	4.52e-03
BMI	0.962	0.936	0.990	6.98e-03
MAP	1.022	1.008	1.035	1.65e-03
e097524	0.861	0.813	0.912	p<0.0001
Age	1.044	1.030	1.059	p<0.0001
Sex	1.307	1.030	1.658	2.77e-02
BMI	0.960	0.934	0.987	3.45e-03
MAP	1.023	1.009	1.036	7.52e-04
e013669	0.885	0.851	0.921	p<0.0001
Age	1.046	1.033	1.060	p<0.0001
Sex	1.377	1.088	1.743	7.73e-03

Predictor	Hazard_Ratio	CI_Lower	CI_Upper	P_Value
BMI	0.960	0.934	0.987	3.93e-03
MAP	1.021	1.008	1.034	1.65e-03
e013736	1.114	1.061	1.169	p<0.0001
Age	1.049	1.035	1.063	p<0.0001
Sex	1.362	1.077	1.722	9.78e-03
BMI	0.960	0.934	0.987	3.98e-03
MAP	1.021	1.008	1.035	1.58e-03
e014441	1.174	1.126	1.223	p<0.0001
Age	1.048	1.034	1.062	p<0.0001
Sex	1.248	0.986	1.581	6.57e-02
BMI	0.958	0.933	0.984	1.89e-03
MAP	1.020	1.008	1.033	1.80e-03
e014598	1.107	1.062	1.154	p<0.0001
Age	1.045	1.032	1.059	p<0.0001
Sex	1.351	1.069	1.709	1.19e-02
BMI	0.962	0.936	0.989	5.56e-03
MAP	1.021	1.008	1.035	1.82e-03
e213095	1.112	1.042	1.187	1.39e-03
Age	1.048	1.034	1.062	p<0.0001
Sex	1.384	1.094	1.751	6.83e-03
BMI	0.961	0.935	0.989	5.76e-03
MAP	1.022	1.008	1.035	1.46e-03
e015362	0.799	0.757	0.844	p<0.0001
Age	1.048	1.034	1.061	p<0.0001
Sex	1.400	1.101	1.780	6.05e-03
BMI	0.968	0.943	0.995	2.01e-02
MAP	1.019	1.006	1.032	4.89e-03
e097631	1.175	1.135	1.218	p<0.0001
Age	1.048	1.034	1.062	p<0.0001
Sex	1.328	1.051	1.679	1.73e-02
BMI	0.958	0.932	0.984	1.65e-03
MAP	1.020	1.007	1.033	1.97e-03
e015560	1.191	1.030	1.377	1.82e-02
Age	1.047	1.033	1.062	p<0.0001
Sex	1.364	1.078	1.727	9.80e-03
BMI	0.962	0.935	0.989	6.17e-03
MAP	1.022	1.008	1.036	1.32e-03
e105680	1.070	1.007	1.136	2.77e-02
Age	1.046	1.032	1.060	p<0.0001
Sex	1.407	1.112	1.781	4.51e-03
BMI	0.962	0.936	0.989	6.68e-03
MAP	1.022	1.008	1.035	1.69e-03
e203120	1.156	1.107	1.208	p<0.0001
Age	1.049	1.035	1.064	p<0.0001
Sex	1.360	1.076	1.719	1.00e-02
BMI	0.964	0.938	0.990	7.86e-03
MAP	1.021	1.009	1.034	1.03e-03
e213326	1.145	1.095	1.197	p<0.0001
Age	1.046	1.033	1.060	p<0.0001
Sex	1.404	1.109	1.776	4.79e-03
BMI	0.958	0.932	0.986	3.05e-03

Predictor	Hazard_Ratio	CI_Lower	CI_Upper	P_Value
MAP	1.021	1.008	1.034	1.42e-03
e017875	1.272	1.183	1.368	p<0.0001
Age	1.046	1.033	1.061	p<0.0001
Sex	1.359	1.076	1.716	9.91e-03
BMI	0.961	0.935	0.988	4.45e-03
MAP	1.020	1.007	1.033	2.79e-03
e017878	1.116	0.967	1.287	1.34e-01
Age	1.048	1.033	1.062	p<0.0001
Sex	1.376	1.088	1.741	7.69e-03
BMI	0.962	0.935	0.989	6.13e-03
MAP	1.022	1.008	1.035	1.64e-03
e105938	1.072	1.015	1.132	1.22e-02
Age	1.047	1.033	1.061	p<0.0001
Sex	1.399	1.107	1.770	4.99e-03
BMI	0.960	0.934	0.988	4.92e-03
MAP	1.022	1.008	1.035	1.49e-03
e018498	0.965	0.899	1.036	3.25e-01
Age	1.047	1.033	1.062	p<0.0001
Sex	1.400	1.104	1.776	5.56e-03
BMI	0.962	0.936	0.990	7.39e-03
MAP	1.021	1.008	1.035	1.83e-03
e208478	1.158	1.056	1.269	1.88e-03
Age	1.048	1.034	1.063	p<0.0001
Sex	1.404	1.109	1.776	4.78e-03
BMI	0.960	0.933	0.988	5.59e-03
MAP	1.022	1.008	1.035	1.52e-03
e018904	1.100	0.975	1.241	1.22e-01
Age	1.048	1.033	1.062	p<0.0001
Sex	1.374	1.086	1.737	7.97e-03
BMI	0.962	0.935	0.989	6.12e-03
MAP	1.022	1.008	1.035	1.66e-03
e019331	1.088	1.035	1.144	1.00e-03
Age	1.046	1.032	1.060	p<0.0001
Sex	1.354	1.070	1.714	1.18e-02
BMI	0.961	0.935	0.989	5.86e-03
MAP	1.022	1.008	1.035	1.51e-03
e019620	1.035	0.976	1.098	2.49e-01
Age	1.047	1.033	1.062	p<0.0001
Sex	1.376	1.088	1.740	7.79e-03
BMI	0.960	0.933	0.988	5.65e-03
MAP	1.022	1.008	1.035	1.47e-03
e019685	1.197	1.158	1.236	p<0.0001
Age	1.042	1.029	1.055	p<0.0001
Sex	1.228	0.970	1.556	8.79e-02
BMI	0.958	0.934	0.983	8.69e-04
MAP	1.020	1.007	1.032	1.99e-03
e019876	1.240	1.163	1.323	p<0.0001
Age	1.046	1.033	1.060	p<0.0001
Sex	1.287	1.017	1.630	3.61e-02
BMI	0.958	0.931	0.984	2.05e-03
MAP	1.021	1.008	1.034	1.90e-03

Predictor	Hazard_Ratio	CI_Lower	CI_Upper	P_Value
e097920	1.329	1.269	1.392	p<0.0001
Age	1.040	1.027	1.053	p<0.0001
Sex	1.216	0.959	1.542	1.07e-01
BMI	0.959	0.935	0.984	1.51e-03
MAP	1.018	1.006	1.030	2.52e-03
e204330	1.375	1.194	1.583	p<0.0001
Age	1.047	1.033	1.061	p<0.0001
Sex	1.386	1.095	1.755	6.60e-03
BMI	0.963	0.937	0.991	9.02e-03
MAP	1.020	1.007	1.034	3.48e-03
e020254	0.998	0.936	1.064	9.46e-01
Age	1.047	1.033	1.062	p<0.0001
Sex	1.384	1.094	1.751	6.72e-03
BMI	0.962	0.936	0.990	7.15e-03
MAP	1.021	1.008	1.035	1.76e-03
e214252	1.109	1.056	1.163	p<0.0001
Age	1.046	1.032	1.060	p<0.0001
Sex	1.332	1.052	1.687	1.75e-02
BMI	0.960	0.934	0.988	4.47e-03
MAP	1.020	1.007	1.034	2.86e-03
e020614	1.117	1.051	1.186	3.54e-04
Age	1.047	1.033	1.062	p<0.0001
Sex	1.334	1.052	1.690	1.73e-02
BMI	0.960	0.933	0.987	3.91e-03
MAP	1.022	1.009	1.036	1.22e-03
e021020	1.150	1.086	1.217	p<0.0001
Age	1.047	1.033	1.061	p<0.0001
Sex	1.342	1.062	1.697	1.38e-02
BMI	0.957	0.931	0.984	2.07e-03
MAP	1.021	1.008	1.034	1.10e-03
e214673	1.015	0.935	1.103	7.19e-01
Age	1.047	1.033	1.062	p<0.0001
Sex	1.382	1.093	1.748	6.98e-03
BMI	0.962	0.936	0.990	7.10e-03
MAP	1.021	1.008	1.035	1.77e-03
e103040	1.146	1.085	1.210	p<0.0001
Age	1.046	1.032	1.060	p<0.0001
Sex	1.387	1.095	1.756	6.60e-03
BMI	0.959	0.933	0.986	2.85e-03
MAP	1.021	1.008	1.034	1.77e-03
e022704	1.342	1.210	1.489	p<0.0001
Age	1.048	1.034	1.062	p<0.0001
Sex	1.351	1.069	1.707	1.18e-02
BMI	0.959	0.933	0.986	2.75e-03
MAP	1.021	1.008	1.034	1.53e-03
e102977	0.985	0.913	1.062	6.94e-01
Age	1.048	1.033	1.062	p<0.0001
Sex	1.382	1.093	1.748	6.82e-03
BMI	0.962	0.935	0.989	6.84e-03
MAP	1.021	1.008	1.035	1.87e-03
e012614	0.868	0.787	0.958	4.77e-03

Predictor	Hazard_Ratio	CI_Lower	CI_Upper	P_Value
Age	1.047	1.033	1.061	p<0.0001
Sex	1.398	1.106	1.768	5.14e-03
BMI	0.964	0.937	0.991	8.75e-03
MAP	1.022	1.008	1.035	1.61e-03
e021136	1.213	1.154	1.275	p<0.0001
Age	1.047	1.033	1.060	p<0.0001
Sex	1.381	1.092	1.748	7.14e-03
BMI	0.958	0.932	0.985	2.30e-03
MAP	1.021	1.008	1.035	1.12e-03
e101808	1.201	1.129	1.278	p<0.0001
Age	1.045	1.032	1.059	p<0.0001
Sex	1.390	1.097	1.763	6.51e-03
BMI	0.963	0.937	0.990	7.99e-03
MAP	1.020	1.007	1.033	2.42e-03

Table S5: Associations between baseline complement peptide intensities and the risk of a CKD event were examined using Cox proportional hazards regression model in training cohort (N=2,774): Model 3

Predictor	Hazard_Ratio	CI_Lower	CI_Upper	P_Value
e300114	0.905	0.792	1.036	1.47e-01
Age	1.022	1.010	1.034	2.16e-04
Sex	1.371	1.076	1.748	1.07e-02
BMI	0.963	0.940	0.986	2.11e-03
MAP	1.017	1.005	1.029	4.93e-03
eGFR	0.970	0.966	0.975	p<0.0001
e209805	0.873	0.740	1.030	1.08e-01
Age	1.022	1.010	1.034	2.48e-04
Sex	1.372	1.076	1.750	1.07e-02
BMI	0.964	0.942	0.988	3.06e-03
MAP	1.017	1.005	1.029	4.44e-03
eGFR	0.970	0.966	0.975	p<0.0001
e004130	0.996	0.885	1.121	9.50e-01
Age	1.022	1.010	1.034	2.75e-04
Sex	1.374	1.078	1.751	1.04e-02
BMI	0.963	0.940	0.987	2.47e-03
MAP	1.017	1.005	1.029	4.45e-03
eGFR	0.970	0.966	0.974	p<0.0001
e005422	0.879	0.801	0.965	6.57e-03
Age	1.023	1.011	1.035	1.35e-04
Sex	1.392	1.093	1.773	7.42e-03
BMI	0.964	0.941	0.987	2.75e-03
MAP	1.016	1.004	1.029	6.97e-03
eGFR	0.970	0.966	0.975	p<0.0001
e207757	0.990	0.948	1.034	6.58e-01
Age	1.021	1.010	1.033	3.61e-04
Sex	1.376	1.079	1.754	1.01e-02
BMI	0.963	0.941	0.987	2.45e-03
MAP	1.017	1.005	1.029	4.43e-03
eGFR	0.970	0.965	0.974	p<0.0001
e007220	0.967	0.885	1.056	4.56e-01
Age	1.022	1.010	1.033	3.16e-04

Predictor	Hazard_Ratio	CI_Lower	CI_Upper	P_Value
Sex	1.379	1.081	1.760	9.79e-03
BMI	0.964	0.941	0.987	2.75e-03
MAP	1.017	1.005	1.029	4.47e-03
eGFR	0.970	0.965	0.974	p<0.0001
e007280	1.029	0.941	1.126	5.31e-01
Age	1.022	1.010	1.034	2.77e-04
Sex	1.364	1.068	1.743	1.28e-02
BMI	0.963	0.940	0.986	2.17e-03
MAP	1.017	1.005	1.029	4.18e-03
eGFR	0.970	0.966	0.975	p<0.0001
e008838	1.059	0.995	1.126	7.10e-02
Age	1.023	1.011	1.035	1.68e-04
Sex	1.358	1.065	1.732	1.35e-02
BMI	0.963	0.940	0.986	2.11e-03
MAP	1.016	1.004	1.028	7.14e-03
eGFR	0.971	0.966	0.976	p<0.0001
e008871	1.012	0.936	1.094	7.63e-01
Age	1.022	1.010	1.034	2.67e-04
Sex	1.374	1.078	1.752	1.03e-02
BMI	0.963	0.940	0.987	2.35e-03
MAP	1.017	1.005	1.029	4.30e-03
eGFR	0.970	0.966	0.975	p<0.0001
e009715	1.032	0.978	1.089	2.51e-01
Age	1.022	1.010	1.034	2.88e-04
Sex	1.365	1.071	1.741	1.20e-02
BMI	0.963	0.940	0.986	2.04e-03
MAP	1.017	1.006	1.029	4.02e-03
eGFR	0.970	0.966	0.975	p<0.0001
e010314	1.033	0.988	1.081	1.57e-01
Age	1.023	1.011	1.036	1.31e-04
Sex	1.360	1.066	1.734	1.33e-02
BMI	0.963	0.940	0.986	2.18e-03
MAP	1.016	1.004	1.028	1.03e-02
eGFR	0.971	0.966	0.976	p<0.0001
e010730	0.933	0.899	0.967	1.85e-04
Age	1.022	1.010	1.034	2.08e-04
Sex	1.347	1.055	1.721	1.70e-02
BMI	0.966	0.943	0.989	4.67e-03
MAP	1.016	1.005	1.028	6.25e-03
eGFR	0.971	0.966	0.975	p<0.0001
e011694	1.045	0.974	1.121	2.17e-01
Age	1.023	1.011	1.035	1.85e-04
Sex	1.351	1.057	1.726	1.63e-02
BMI	0.963	0.940	0.986	2.10e-03
MAP	1.017	1.005	1.029	4.90e-03
eGFR	0.971	0.966	0.975	p<0.0001
e011859	0.994	0.930	1.063	8.63e-01
Age	1.022	1.010	1.034	2.82e-04
Sex	1.376	1.079	1.755	1.02e-02
BMI	0.963	0.940	0.987	2.48e-03
MAP	1.017	1.005	1.029	4.58e-03

Predictor	Hazard_Ratio	CI_Lower	CI_Upper	P_Value
eGFR	0.970	0.965	0.974	p<0.0001
e012507	1.024	0.974	1.076	3.57e-01
Age	1.022	1.010	1.034	2.43e-04
Sex	1.380	1.082	1.759	9.43e-03
BMI	0.964	0.941	0.987	2.43e-03
MAP	1.017	1.005	1.029	4.16e-03
eGFR	0.970	0.966	0.974	p<0.0001
e212456	1.063	1.016	1.111	7.72e-03
Age	1.022	1.010	1.034	2.29e-04
Sex	1.350	1.057	1.726	1.64e-02
BMI	0.963	0.940	0.986	1.89e-03
MAP	1.017	1.006	1.029	3.62e-03
eGFR	0.971	0.966	0.975	p<0.0001
e013024	0.891	0.816	0.972	9.51e-03
Age	1.021	1.009	1.032	5.11e-04
Sex	1.402	1.100	1.787	6.32e-03
BMI	0.963	0.940	0.986	2.04e-03
MAP	1.017	1.005	1.029	4.77e-03
eGFR	0.970	0.965	0.974	p<0.0001
e097524	0.959	0.905	1.016	1.58e-01
Age	1.022	1.010	1.034	2.81e-04
Sex	1.344	1.049	1.722	1.94e-02
BMI	0.962	0.939	0.986	1.65e-03
MAP	1.018	1.006	1.030	3.27e-03
eGFR	0.971	0.966	0.975	p<0.0001
e013669	0.920	0.883	0.959	p<0.0001
Age	1.022	1.011	1.034	1.81e-04
Sex	1.397	1.093	1.785	7.55e-03
BMI	0.962	0.939	0.986	1.75e-03
MAP	1.017	1.005	1.028	4.48e-03
eGFR	0.971	0.967	0.976	p<0.0001
e013736	1.014	0.967	1.064	5.71e-01
Age	1.022	1.010	1.035	2.85e-04
Sex	1.366	1.070	1.744	1.22e-02
BMI	0.963	0.940	0.987	2.24e-03
MAP	1.017	1.005	1.029	4.75e-03
eGFR	0.970	0.966	0.975	p<0.0001
e014441	1.042	0.996	1.091	7.64e-02
Age	1.024	1.012	1.036	1.08e-04
Sex	1.330	1.041	1.699	2.26e-02
BMI	0.962	0.939	0.986	1.69e-03
MAP	1.017	1.005	1.028	5.69e-03
eGFR	0.972	0.967	0.977	p<0.0001
e014598	1.049	1.006	1.094	2.45e-02
Age	1.022	1.010	1.034	2.89e-04
Sex	1.366	1.070	1.743	1.22e-02
BMI	0.962	0.940	0.986	1.80e-03
MAP	1.018	1.006	1.030	3.86e-03
eGFR	0.971	0.966	0.976	p<0.0001
e213095	0.999	0.940	1.063	9.79e-01
Age	1.022	1.010	1.034	3.45e-04

Predictor	Hazard_Ratio	CI_Lower	CI_Upper	P_Value
Sex	1.374	1.078	1.752	1.04e-02
BMI	0.963	0.940	0.987	2.31e-03
MAP	1.017	1.005	1.029	4.46e-03
eGFR	0.970	0.965	0.975	p<0.0001
e015362	0.892	0.841	0.947	1.89e-04
Age	1.025	1.013	1.037	p<0.0001
Sex	1.372	1.074	1.752	1.13e-02
BMI	0.967	0.944	0.990	6.09e-03
MAP	1.016	1.004	1.028	8.42e-03
eGFR	0.973	0.969	0.978	p<0.0001
e097631	1.061	1.017	1.106	5.73e-03
Age	1.024	1.012	1.036	1.10e-04
Sex	1.322	1.036	1.687	2.47e-02
BMI	0.962	0.939	0.985	1.47e-03
MAP	1.016	1.005	1.028	6.45e-03
eGFR	0.972	0.967	0.977	p<0.0001
e015560	1.025	0.886	1.186	7.40e-01
Age	1.022	1.010	1.034	2.57e-04
Sex	1.370	1.073	1.749	1.16e-02
BMI	0.963	0.940	0.987	2.29e-03
MAP	1.017	1.005	1.029	4.36e-03
eGFR	0.970	0.966	0.974	p<0.0001
e105680	1.033	0.968	1.102	3.25e-01
Age	1.021	1.009	1.033	4.16e-04
Sex	1.384	1.084	1.769	9.25e-03
BMI	0.964	0.941	0.987	2.58e-03
MAP	1.017	1.005	1.029	4.68e-03
eGFR	0.970	0.966	0.975	p<0.0001
e203120	1.071	1.025	1.118	2.14e-03
Age	1.025	1.012	1.037	p<0.0001
Sex	1.351	1.059	1.722	1.53e-02
BMI	0.963	0.940	0.987	2.29e-03
MAP	1.017	1.005	1.028	4.81e-03
eGFR	0.972	0.967	0.976	p<0.0001
e213326	1.051	1.007	1.096	2.28e-02
Age	1.023	1.011	1.035	1.63e-04
Sex	1.369	1.073	1.745	1.15e-02
BMI	0.962	0.939	0.986	1.91e-03
MAP	1.017	1.005	1.029	4.43e-03
eGFR	0.972	0.967	0.976	p<0.0001
e017875	1.089	1.010	1.173	2.62e-02
Age	1.022	1.010	1.034	2.34e-04
Sex	1.362	1.070	1.735	1.22e-02
BMI	0.963	0.940	0.986	1.92e-03
MAP	1.016	1.004	1.028	6.84e-03
eGFR	0.971	0.966	0.975	p<0.0001
e017878	1.017	0.894	1.156	8.03e-01
Age	1.022	1.010	1.034	2.61e-04
Sex	1.371	1.074	1.750	1.12e-02
BMI	0.963	0.940	0.987	2.19e-03
MAP	1.017	1.005	1.029	4.43e-03

Predictor	Hazard_Ratio	CI_Lower	CI_Upper	P_Value
eGFR	0.970	0.966	0.974	p<0.0001
e105938	1.071	1.012	1.133	1.81e-02
Age	1.021	1.009	1.033	4.06e-04
Sex	1.392	1.090	1.778	8.00e-03
BMI	0.961	0.938	0.985	1.49e-03
MAP	1.017	1.005	1.029	3.97e-03
eGFR	0.970	0.966	0.974	p<0.0001
e018498	0.955	0.892	1.023	1.94e-01
Age	1.022	1.010	1.034	2.51e-04
Sex	1.385	1.085	1.768	8.89e-03
BMI	0.963	0.941	0.987	2.40e-03
MAP	1.017	1.005	1.029	4.78e-03
eGFR	0.970	0.965	0.974	p<0.0001
e208478	1.097	0.995	1.209	6.18e-02
Age	1.023	1.011	1.035	2.03e-04
Sex	1.371	1.075	1.749	1.09e-02
BMI	0.963	0.940	0.987	2.41e-03
MAP	1.017	1.005	1.029	4.65e-03
eGFR	0.970	0.966	0.975	p<0.0001
e018904	0.966	0.849	1.098	5.94e-01
Age	1.022	1.010	1.034	3.10e-04
Sex	1.377	1.080	1.756	9.80e-03
BMI	0.963	0.941	0.987	2.43e-03
MAP	1.017	1.005	1.029	4.54e-03
eGFR	0.970	0.965	0.974	p<0.0001
e019331	1.062	1.013	1.112	1.19e-02
Age	1.021	1.009	1.033	4.55e-04
Sex	1.350	1.057	1.725	1.63e-02
BMI	0.963	0.940	0.987	2.34e-03
MAP	1.017	1.005	1.029	3.98e-03
eGFR	0.970	0.966	0.975	p<0.0001
e019620	0.999	0.949	1.052	9.77e-01
Age	1.022	1.010	1.034	2.71e-04
Sex	1.374	1.077	1.752	1.05e-02
BMI	0.963	0.940	0.987	2.99e-03
MAP	1.017	1.005	1.029	4.44e-03
eGFR	0.970	0.966	0.974	p<0.0001
e019685	1.076	1.032	1.122	6.52e-04
Age	1.024	1.013	1.036	p<0.0001
Sex	1.294	1.013	1.652	3.93e-02
BMI	0.962	0.939	0.985	1.14e-03
MAP	1.017	1.005	1.029	4.07e-03
eGFR	0.975	0.969	0.981	p<0.0001
e019876	1.096	1.028	1.169	5.12e-03
Age	1.023	1.011	1.035	1.27e-04
Sex	1.323	1.035	1.690	2.52e-02
BMI	0.960	0.937	0.984	1.19e-03
MAP	1.017	1.005	1.029	4.89e-03
eGFR	0.972	0.967	0.976	p<0.0001
e097920	1.182	1.118	1.249	p<0.0001
Age	1.024	1.012	1.037	p<0.0001

Predictor	Hazard_Ratio	CI_Lower	CI_Upper	P_Value
Sex	1.269	0.991	1.625	5.95e-02
BMI	0.961	0.938	0.984	1.20e-03
MAP	1.016	1.004	1.027	6.92e-03
eGFR	0.978	0.973	0.984	p<0.0001
e204330	1.305	1.145	1.487	p<0.0001
Age	1.022	1.010	1.034	2.90e-04
Sex	1.377	1.080	1.756	9.88e-03
BMI	0.964	0.941	0.987	2.84e-03
MAP	1.016	1.004	1.028	7.82e-03
eGFR	0.970	0.966	0.975	p<0.0001
e020254	0.964	0.908	1.024	2.37e-01
Age	1.021	1.009	1.033	4.66e-04
Sex	1.379	1.081	1.759	9.56e-03
BMI	0.963	0.940	0.986	1.95e-03
MAP	1.018	1.006	1.030	3.59e-03
eGFR	0.970	0.965	0.974	p<0.0001
e214252	1.031	0.986	1.077	1.84e-01
Age	1.022	1.011	1.034	1.83e-04
Sex	1.347	1.053	1.722	1.77e-02
BMI	0.963	0.941	0.987	2.33e-03
MAP	1.016	1.005	1.028	6.38e-03
eGFR	0.971	0.966	0.975	p<0.0001
e020614	0.969	0.909	1.033	3.32e-01
Age	1.021	1.009	1.033	4.19e-04
Sex	1.392	1.089	1.778	8.26e-03
BMI	0.964	0.941	0.987	2.81e-03
MAP	1.017	1.005	1.029	5.03e-03
eGFR	0.969	0.965	0.974	p<0.0001
e021020	1.022	0.964	1.084	4.68e-01
Age	1.022	1.010	1.034	2.45e-04
Sex	1.360	1.065	1.737	1.37e-02
BMI	0.963	0.940	0.986	1.96e-03
MAP	1.017	1.005	1.029	4.29e-03
eGFR	0.970	0.966	0.975	p<0.0001
e214673	1.020	0.939	1.108	6.40e-01
Age	1.022	1.010	1.034	2.65e-04
Sex	1.370	1.074	1.746	1.11e-02
BMI	0.963	0.940	0.987	2.30e-03
MAP	1.017	1.005	1.029	4.39e-03
eGFR	0.970	0.966	0.974	p<0.0001
e103040	1.078	1.025	1.135	3.92e-03
Age	1.021	1.010	1.033	3.73e-04
Sex	1.351	1.059	1.722	1.54e-02
BMI	0.961	0.938	0.985	1.31e-03
MAP	1.017	1.005	1.029	5.20e-03
eGFR	0.971	0.967	0.976	p<0.0001
e022704	1.100	0.982	1.232	9.94e-02
Age	1.023	1.011	1.035	1.36e-04
Sex	1.361	1.068	1.734	1.28e-02
BMI	0.962	0.939	0.985	1.61e-03
MAP	1.017	1.005	1.029	5.20e-03

Predictor	Hazard_Ratio	CI_Lower	CI_Upper	P_Value
eGFR	0.971	0.966	0.975	p<0.0001
e102977	0.977	0.905	1.054	5.45e-01
Age	1.022	1.010	1.034	2.41e-04
Sex	1.372	1.077	1.748	1.05e-02
BMI	0.963	0.940	0.986	2.10e-03
MAP	1.017	1.005	1.029	5.07e-03
eGFR	0.970	0.966	0.974	p<0.0001
e012614	0.898	0.817	0.987	2.54e-02
Age	1.021	1.010	1.033	3.54e-04
Sex	1.381	1.083	1.761	9.22e-03
BMI	0.964	0.941	0.987	2.38e-03
MAP	1.017	1.005	1.029	4.34e-03
eGFR	0.970	0.966	0.975	p<0.0001
e021136	1.066	1.011	1.125	1.85e-02
Age	1.024	1.012	1.036	1.03e-04
Sex	1.352	1.061	1.724	1.49e-02
BMI	0.962	0.939	0.986	1.96e-03
MAP	1.017	1.005	1.029	4.72e-03
eGFR	0.972	0.967	0.978	p<0.0001
e101808	1.082	1.013	1.155	1.88e-02
Age	1.022	1.010	1.034	2.62e-04
Sex	1.373	1.074	1.754	1.13e-02
BMI	0.964	0.941	0.988	3.25e-03
MAP	1.016	1.005	1.028	6.11e-03
eGFR	0.971	0.967	0.976	p<0.0001

Table S6: Associations between baseline complement peptide intensities and the risk of a CKD event were examined using Cox proportional hazards regression model in training cohort (N=2,774): Model 4

Predictor	Hazard_Ratio	CI_Lower	CI_Upper	P_Value
e300114	0.958	0.837	1.095	5.28e-01
Age	1.039	1.027	1.051	p<0.0001
Sex	1.121	0.877	1.432	3.62e-01
BMI	0.961	0.940	0.982	3.39e-04
MAP	1.011	0.999	1.022	6.88e-02
eGFR	0.991	0.985	0.998	7.65e-03
Albumin_KreatininQuotient	1.464	1.368	1.568	p<0.0001
e209805	0.875	0.740	1.033	1.15e-01
Age	1.039	1.027	1.051	p<0.0001
Sex	1.117	0.874	1.428	3.76e-01
BMI	0.962	0.941	0.983	5.24e-04
MAP	1.011	0.999	1.023	6.38e-02
eGFR	0.992	0.985	0.998	1.10e-02
Albumin_KreatininQuotient	1.468	1.372	1.571	p<0.0001
e004130	1.001	0.895	1.119	9.91e-01
Age	1.039	1.027	1.051	p<0.0001
Sex	1.119	0.876	1.430	3.68e-01
BMI	0.961	0.940	0.982	3.57e-04
MAP	1.011	0.999	1.023	6.51e-02
eGFR	0.991	0.985	0.998	7.52e-03

Predictor	Hazard_Ratio	CI_Lower	CI_Upper	P_Value
Albumin_KreatininQuotient	1.467	1.371	1.571	p<0.0001
e005422	0.926	0.849	1.011	8.55e-02
Age	1.039	1.027	1.051	p<0.0001
Sex	1.134	0.888	1.449	3.14e-01
BMI	0.961	0.940	0.982	3.86e-04
MAP	1.011	0.999	1.022	7.64e-02
eGFR	0.991	0.985	0.997	6.17e-03
Albumin_KreatininQuotient	1.455	1.358	1.559	p<0.0001
e207757	0.937	0.898	0.978	2.78e-03
Age	1.037	1.025	1.049	p<0.0001
Sex	1.127	0.881	1.442	3.40e-01
BMI	0.962	0.941	0.983	5.03e-04
MAP	1.011	0.999	1.023	6.55e-02
eGFR	0.990	0.984	0.997	2.80e-03
Albumin_KreatininQuotient	1.496	1.399	1.601	p<0.0001
e007220	0.969	0.889	1.057	4.81e-01
Age	1.039	1.027	1.051	p<0.0001
Sex	1.120	0.877	1.432	3.64e-01
BMI	0.961	0.940	0.982	4.04e-04
MAP	1.011	0.999	1.022	6.84e-02
eGFR	0.991	0.985	0.998	6.70e-03
Albumin_KreatininQuotient	1.467	1.371	1.570	p<0.0001
e007280	1.004	0.922	1.093	9.30e-01
Age	1.039	1.027	1.051	p<0.0001
Sex	1.118	0.874	1.431	3.75e-01
BMI	0.961	0.940	0.982	3.41e-04
MAP	1.011	0.999	1.023	6.72e-02
eGFR	0.991	0.985	0.998	8.59e-03
Albumin_KreatininQuotient	1.467	1.371	1.570	p<0.0001
e008838	0.953	0.895	1.015	1.37e-01
Age	1.039	1.027	1.051	p<0.0001
Sex	1.117	0.875	1.426	3.73e-01
BMI	0.960	0.940	0.982	3.08e-04
MAP	1.011	1.000	1.023	5.66e-02
eGFR	0.991	0.985	0.997	6.26e-03
Albumin_KreatininQuotient	1.489	1.389	1.596	p<0.0001
e008871	0.977	0.908	1.051	5.33e-01
Age	1.039	1.027	1.051	p<0.0001
Sex	1.119	0.876	1.430	3.69e-01
BMI	0.961	0.940	0.982	3.35e-04
MAP	1.011	0.999	1.023	6.85e-02
eGFR	0.991	0.984	0.997	6.88e-03
Albumin_KreatininQuotient	1.469	1.373	1.572	p<0.0001
e009715	0.992	0.940	1.047	7.77e-01
Age	1.039	1.027	1.051	p<0.0001
Sex	1.120	0.876	1.431	3.65e-01
BMI	0.961	0.940	0.982	3.78e-04
MAP	1.011	0.999	1.023	6.98e-02
eGFR	0.991	0.985	0.998	7.82e-03
Albumin_KreatininQuotient	1.469	1.372	1.574	p<0.0001
e010314	0.951	0.908	0.997	3.63e-02

Predictor	Hazard_Ratio	CI_Lower	CI_Upper	P_Value
Age	1.037	1.025	1.049	p<0.0001
Sex	1.125	0.882	1.435	3.44e-01
BMI	0.961	0.940	0.982	3.15e-04
MAP	1.012	1.000	1.024	4.36e-02
eGFR	0.991	0.985	0.997	5.96e-03
Albumin_KreatininQuotient	1.504	1.400	1.616	p<0.0001
e010730	0.948	0.913	0.983	4.55e-03
Age	1.039	1.027	1.051	p<0.0001
Sex	1.110	0.867	1.421	4.09e-01
BMI	0.963	0.942	0.984	7.67e-04
MAP	1.010	0.999	1.022	8.56e-02
eGFR	0.991	0.984	0.997	5.56e-03
Albumin_KreatininQuotient	1.448	1.351	1.552	p<0.0001
e011694	0.951	0.886	1.021	1.68e-01
Age	1.038	1.026	1.050	p<0.0001
Sex	1.134	0.887	1.452	3.16e-01
BMI	0.961	0.940	0.983	4.06e-04
MAP	1.011	0.999	1.023	6.98e-02
eGFR	0.991	0.985	0.998	6.92e-03
Albumin_KreatininQuotient	1.483	1.383	1.590	p<0.0001
e011859	0.931	0.872	0.994	3.12e-02
Age	1.039	1.027	1.051	p<0.0001
Sex	1.130	0.885	1.444	3.28e-01
BMI	0.962	0.941	0.983	5.02e-04
MAP	1.010	0.998	1.021	9.92e-02
eGFR	0.991	0.984	0.997	5.43e-03
Albumin_KreatininQuotient	1.489	1.390	1.594	p<0.0001
e012507	1.031	0.982	1.083	2.16e-01
Age	1.039	1.027	1.051	p<0.0001
Sex	1.127	0.882	1.440	3.40e-01
BMI	0.961	0.940	0.982	3.40e-04
MAP	1.011	0.999	1.023	6.40e-02
eGFR	0.991	0.985	0.998	7.01e-03
Albumin_KreatininQuotient	1.468	1.372	1.571	p<0.0001
e212456	1.043	0.999	1.090	5.75e-02
Age	1.039	1.027	1.051	p<0.0001
Sex	1.107	0.866	1.416	4.17e-01
BMI	0.960	0.939	0.981	2.54e-04
MAP	1.011	1.000	1.023	5.78e-02
eGFR	0.991	0.985	0.998	9.55e-03
Albumin_KreatininQuotient	1.458	1.361	1.563	p<0.0001
e013024	0.867	0.796	0.943	9.24e-04
Age	1.038	1.026	1.050	p<0.0001
Sex	1.145	0.895	1.463	2.81e-01
BMI	0.960	0.939	0.981	2.53e-04
MAP	1.010	0.999	1.022	8.04e-02
eGFR	0.991	0.984	0.997	4.55e-03
Albumin_KreatininQuotient	1.478	1.381	1.582	p<0.0001
e097524	1.006	0.951	1.066	8.25e-01
Age	1.039	1.027	1.051	p<0.0001
Sex	1.122	0.876	1.439	3.62e-01

Predictor	Hazard_Ratio	CI_Lower	CI_Upper	P_Value
BMI	0.961	0.940	0.982	3.52e-04
MAP	1.011	0.999	1.023	6.96e-02
eGFR	0.991	0.985	0.998	7.21e-03
Albumin_KreatininQuotient	1.468	1.371	1.572	p<0.0001
e013669	0.962	0.921	1.004	7.40e-02
Age	1.039	1.026	1.051	p<0.0001
Sex	1.126	0.879	1.441	3.48e-01
BMI	0.960	0.939	0.982	3.53e-04
MAP	1.011	0.999	1.022	7.35e-02
eGFR	0.991	0.985	0.998	6.76e-03
Albumin_KreatininQuotient	1.447	1.348	1.553	p<0.0001
e013736	0.943	0.900	0.989	1.52e-02
Age	1.037	1.025	1.050	p<0.0001
Sex	1.139	0.889	1.458	3.02e-01
BMI	0.961	0.940	0.982	3.70e-04
MAP	1.010	0.999	1.022	8.50e-02
eGFR	0.991	0.985	0.997	6.28e-03
Albumin_KreatininQuotient	1.500	1.399	1.607	p<0.0001
e014441	0.945	0.904	0.988	1.24e-02
Age	1.038	1.026	1.050	p<0.0001
Sex	1.147	0.897	1.467	2.74e-01
BMI	0.962	0.941	0.983	4.81e-04
MAP	1.011	1.000	1.023	5.86e-02
eGFR	0.991	0.984	0.997	3.80e-03
Albumin_KreatininQuotient	1.512	1.411	1.621	p<0.0001
e014598	1.054	1.011	1.099	1.40e-02
Age	1.039	1.027	1.051	p<0.0001
Sex	1.118	0.875	1.429	3.72e-01
BMI	0.960	0.939	0.981	2.19e-04
MAP	1.012	1.000	1.024	4.28e-02
eGFR	0.992	0.986	0.999	2.42e-02
Albumin_KreatininQuotient	1.472	1.375	1.577	p<0.0001
e213095	0.947	0.892	1.005	7.45e-02
Age	1.038	1.026	1.050	p<0.0001
Sex	1.126	0.881	1.440	3.42e-01
BMI	0.961	0.940	0.982	3.60e-04
MAP	1.010	0.999	1.022	7.85e-02
eGFR	0.991	0.984	0.997	5.26e-03
Albumin_KreatininQuotient	1.480	1.383	1.584	p<0.0001
e015362	0.973	0.910	1.041	4.33e-01
Age	1.039	1.027	1.052	p<0.0001
Sex	1.124	0.879	1.437	3.53e-01
BMI	0.961	0.940	0.983	4.90e-04
MAP	1.011	0.999	1.022	7.18e-02
eGFR	0.992	0.985	0.998	1.13e-02
Albumin_KreatininQuotient	1.454	1.353	1.563	p<0.0001
e097631	0.987	0.947	1.028	5.27e-01
Age	1.039	1.027	1.051	p<0.0001
Sex	1.124	0.879	1.437	3.53e-01
BMI	0.961	0.940	0.982	3.44e-04
MAP	1.011	0.999	1.023	6.24e-02

Predictor	Hazard_Ratio	CI_Lower	CI_Upper	P_Value
eGFR	0.991	0.984	0.997	6.71e-03
Albumin_KreatininQuotient	1.475	1.376	1.581	p<0.0001
e015560	0.948	0.820	1.095	4.67e-01
Age	1.039	1.027	1.051	p<0.0001
Sex	1.126	0.881	1.440	3.44e-01
BMI	0.961	0.940	0.982	3.59e-04
MAP	1.011	0.999	1.022	7.56e-02
eGFR	0.991	0.985	0.998	7.00e-03
Albumin_KreatininQuotient	1.470	1.374	1.573	p<0.0001
e105680	1.085	1.018	1.157	1.17e-02
Age	1.038	1.026	1.050	p<0.0001
Sex	1.149	0.896	1.473	2.73e-01
BMI	0.962	0.941	0.983	4.83e-04
MAP	1.012	1.000	1.023	5.22e-02
eGFR	0.992	0.986	0.998	1.40e-02
Albumin_KreatininQuotient	1.484	1.386	1.588	p<0.0001
e203120	1.022	0.981	1.065	2.98e-01
Age	1.039	1.027	1.052	p<0.0001
Sex	1.121	0.878	1.432	3.60e-01
BMI	0.960	0.939	0.982	3.44e-04
MAP	1.011	0.999	1.022	6.81e-02
eGFR	0.992	0.985	0.998	1.07e-02
Albumin_KreatininQuotient	1.458	1.362	1.562	p<0.0001
e213326	0.995	0.956	1.036	8.19e-01
Age	1.039	1.027	1.051	p<0.0001
Sex	1.118	0.875	1.429	3.73e-01
BMI	0.961	0.940	0.982	3.42e-04
MAP	1.011	0.999	1.023	6.57e-02
eGFR	0.991	0.985	0.998	7.43e-03
Albumin_KreatininQuotient	1.470	1.371	1.575	p<0.0001
e017875	1.003	0.925	1.086	9.51e-01
Age	1.039	1.027	1.051	p<0.0001
Sex	1.119	0.876	1.430	3.68e-01
BMI	0.961	0.940	0.982	3.35e-04
MAP	1.011	0.999	1.023	6.59e-02
eGFR	0.991	0.985	0.998	7.60e-03
Albumin_KreatininQuotient	1.467	1.370	1.570	p<0.0001
e017878	0.943	0.833	1.067	3.50e-01
Age	1.039	1.027	1.051	p<0.0001
Sex	1.124	0.879	1.437	3.51e-01
BMI	0.961	0.940	0.983	4.50e-04
MAP	1.011	0.999	1.022	6.74e-02
eGFR	0.991	0.985	0.998	6.88e-03
Albumin_KreatininQuotient	1.470	1.374	1.574	p<0.0001
e105938	1.082	1.023	1.145	5.79e-03
Age	1.039	1.027	1.051	p<0.0001
Sex	1.141	0.892	1.459	2.94e-01
BMI	0.958	0.937	0.980	1.59e-04
MAP	1.011	1.000	1.023	4.90e-02
eGFR	0.991	0.985	0.998	8.14e-03
Albumin_KreatininQuotient	1.473	1.376	1.577	p<0.0001

Predictor	Hazard_Ratio	CI_Lower	CI_Upper	P_Value
e018498	0.977	0.914	1.045	5.05e-01
Age	1.039	1.027	1.051	p<0.0001
Sex	1.126	0.879	1.441	3.48e-01
BMI	0.961	0.940	0.982	3.63e-04
MAP	1.011	0.999	1.022	6.93e-02
eGFR	0.991	0.985	0.998	7.22e-03
Albumin_KreatininQuotient	1.464	1.367	1.568	p<0.0001
e208478	1.069	0.968	1.181	1.90e-01
Age	1.040	1.027	1.052	p<0.0001
Sex	1.118	0.875	1.429	3.71e-01
BMI	0.960	0.939	0.982	3.52e-04
MAP	1.011	0.999	1.023	6.54e-02
eGFR	0.991	0.985	0.998	9.18e-03
Albumin_KreatininQuotient	1.465	1.368	1.569	p<0.0001
e018904	0.877	0.769	0.999	4.78e-02
Age	1.039	1.027	1.051	p<0.0001
Sex	1.133	0.886	1.447	3.19e-01
BMI	0.961	0.941	0.983	4.06e-04
MAP	1.010	0.999	1.022	7.62e-02
eGFR	0.991	0.985	0.998	7.40e-03
Albumin_KreatininQuotient	1.481	1.383	1.586	p<0.0001
e019331	1.096	1.046	1.148	1.24e-04
Age	1.039	1.027	1.051	p<0.0001
Sex	1.088	0.851	1.390	5.02e-01
BMI	0.959	0.938	0.980	2.23e-04
MAP	1.012	1.001	1.024	3.62e-02
eGFR	0.993	0.987	1.000	4.04e-02
Albumin_KreatininQuotient	1.503	1.401	1.613	p<0.0001
e019620	0.962	0.916	1.010	1.15e-01
Age	1.039	1.027	1.051	p<0.0001
Sex	1.121	0.878	1.432	3.59e-01
BMI	0.963	0.942	0.984	7.68e-04
MAP	1.011	0.999	1.023	6.83e-02
eGFR	0.991	0.985	0.997	6.09e-03
Albumin_KreatininQuotient	1.477	1.380	1.580	p<0.0001
e019685	0.952	0.911	0.994	2.67e-02
Age	1.040	1.028	1.052	p<0.0001
Sex	1.137	0.891	1.452	3.03e-01
BMI	0.962	0.941	0.983	5.04e-04
MAP	1.010	0.999	1.022	8.36e-02
eGFR	0.990	0.984	0.997	3.80e-03
Albumin_KreatininQuotient	1.537	1.424	1.659	p<0.0001
e019876	1.033	0.973	1.098	2.90e-01
Age	1.039	1.027	1.052	p<0.0001
Sex	1.111	0.870	1.421	3.99e-01
BMI	0.960	0.939	0.981	2.82e-04
MAP	1.011	0.999	1.023	6.38e-02
eGFR	0.991	0.985	0.998	9.31e-03
Albumin_KreatininQuotient	1.458	1.361	1.562	p<0.0001
e097920	1.104	1.044	1.167	5.09e-04
Age	1.039	1.026	1.051	p<0.0001

Predictor	Hazard_Ratio	CI_Lower	CI_Upper	P_Value
Sex	1.101	0.860	1.410	4.43e-01
BMI	0.960	0.939	0.982	3.38e-04
MAP	1.011	0.999	1.022	6.72e-02
eGFR	0.994	0.987	1.001	8.93e-02
Albumin_KreatininQuotient	1.418	1.320	1.524	p<0.0001
e204330	1.184	1.019	1.375	2.72e-02
Age	1.038	1.026	1.051	p<0.0001
Sex	1.134	0.887	1.451	3.15e-01
BMI	0.960	0.939	0.982	3.65e-04
MAP	1.010	0.999	1.022	7.95e-02
eGFR	0.991	0.985	0.997	5.95e-03
Albumin_KreatininQuotient	1.457	1.361	1.560	p<0.0001
e020254	0.950	0.895	1.008	8.78e-02
Age	1.038	1.026	1.050	p<0.0001
Sex	1.132	0.885	1.447	3.24e-01
BMI	0.960	0.940	0.981	2.74e-04
MAP	1.011	1.000	1.023	5.67e-02
eGFR	0.991	0.984	0.997	5.60e-03
Albumin_KreatininQuotient	1.470	1.373	1.574	p<0.0001
e214252	0.973	0.932	1.017	2.24e-01
Age	1.039	1.027	1.051	p<0.0001
Sex	1.138	0.887	1.459	3.09e-01
BMI	0.961	0.940	0.982	3.17e-04
MAP	1.011	1.000	1.023	5.70e-02
eGFR	0.991	0.985	0.997	6.08e-03
Albumin_KreatininQuotient	1.482	1.383	1.589	p<0.0001
e020614	0.902	0.849	0.959	9.85e-04
Age	1.038	1.026	1.050	p<0.0001
Sex	1.164	0.909	1.492	2.29e-01
BMI	0.963	0.942	0.984	5.83e-04
MAP	1.010	0.998	1.021	1.08e-01
eGFR	0.990	0.984	0.997	3.17e-03
Albumin_KreatininQuotient	1.499	1.402	1.603	p<0.0001
e021020	0.943	0.890	0.998	4.23e-02
Age	1.038	1.026	1.050	p<0.0001
Sex	1.132	0.885	1.449	3.23e-01
BMI	0.963	0.943	0.985	8.14e-04
MAP	1.010	0.999	1.022	8.54e-02
eGFR	0.991	0.984	0.997	4.09e-03
Albumin_KreatininQuotient	1.492	1.393	1.597	p<0.0001
e214673	1.013	0.932	1.100	7.68e-01
Age	1.039	1.027	1.051	p<0.0001
Sex	1.119	0.876	1.429	3.69e-01
BMI	0.961	0.940	0.982	3.38e-04
MAP	1.011	0.999	1.023	6.46e-02
eGFR	0.991	0.985	0.998	7.72e-03
Albumin_KreatininQuotient	1.467	1.371	1.571	p<0.0001
e103040	1.066	1.016	1.119	9.29e-03
Age	1.038	1.026	1.051	p<0.0001
Sex	1.107	0.868	1.411	4.12e-01
BMI	0.958	0.938	0.980	1.62e-04

Predictor	Hazard_Ratio	CI_Lower	CI_Upper	P_Value
MAP	1.011	1.000	1.023	5.90e-02
eGFR	0.992	0.985	0.998	1.42e-02
Albumin_KreatininQuotient	1.464	1.368	1.567	p<0.0001
e022704	1.050	0.935	1.179	4.07e-01
Age	1.039	1.027	1.052	p<0.0001
Sex	1.118	0.875	1.429	3.73e-01
BMI	0.960	0.939	0.981	2.88e-04
MAP	1.011	0.999	1.022	7.14e-02
eGFR	0.992	0.985	0.998	1.14e-02
Albumin_KreatininQuotient	1.465	1.368	1.568	p<0.0001
e102977	1.000	0.924	1.081	9.91e-01
Age	1.039	1.027	1.051	p<0.0001
Sex	1.119	0.876	1.429	3.68e-01
BMI	0.961	0.940	0.982	3.73e-04
MAP	1.011	0.999	1.023	6.62e-02
eGFR	0.991	0.985	0.998	7.88e-03
Albumin_KreatininQuotient	1.467	1.370	1.571	p<0.0001
e012614	0.913	0.834	1.000	5.03e-02
Age	1.038	1.026	1.050	p<0.0001
Sex	1.121	0.877	1.432	3.62e-01
BMI	0.960	0.939	0.982	2.86e-04
MAP	1.011	0.999	1.022	7.01e-02
eGFR	0.991	0.985	0.997	6.47e-03
Albumin_KreatininQuotient	1.462	1.366	1.565	p<0.0001
e021136	0.991	0.943	1.043	7.35e-01
Age	1.039	1.027	1.051	p<0.0001
Sex	1.118	0.875	1.428	3.73e-01
BMI	0.961	0.940	0.982	3.28e-04
MAP	1.011	0.999	1.022	6.64e-02
eGFR	0.991	0.984	0.998	8.17e-03
Albumin_KreatininQuotient	1.471	1.373	1.576	p<0.0001
e101808	1.063	0.997	1.132	6.05e-02
Age	1.039	1.027	1.051	p<0.0001
Sex	1.112	0.870	1.421	3.97e-01
BMI	0.961	0.940	0.982	3.85e-04
MAP	1.011	1.000	1.022	5.92e-02
eGFR	0.992	0.986	0.999	1.66e-02
Albumin_KreatininQuotient	1.464	1.368	1.568	p<0.0001
